# Striatal dopamine gene network moderates the effect of early adversity on the risk for adult psychiatric and cardiometabolic comorbidity

**DOI:** 10.1038/s41598-024-78465-5

**Published:** 2024-11-09

**Authors:** Barbara Barth, Danusa Mar Arcego, Euclides José de Mendonça Filho, Randriely Merscher Sobreira de Lima, Carine Parent, Carla Dalmaz, André Krumel Portella, Irina Pokhvisneva, Michael J. Meaney, Patricia Pelufo Silveira

**Affiliations:** 1 Integrated Program in Neurosciences, McGill University, Montreal, QC Canada; 2 Douglas Mental Health University Institute, McGill University, Montreal, QC Canada; 3 Ludmer Centre for Neuroinformatics and Mental Health, Douglas Research Centre, McGill University, Montreal, QC Canada; 4 Programa de Pós-Graduação em Neurociências, Instituto de Ciências Básicas da Saúde (ICBS), Universidade Federal do Rio Grande do Sul, Porto Alegre, RS Brazil; 5 Department of Psychiatry, Faculty of Medicine and Health Sciences, McGill University, Montreal, QC Canada; 6 Department of Paediatrics, Yong Lin Loon School of Medicine, National University of Singapore, Singapore, Singapore; 7 Translational Neuroscience Programme, Singapore Institute for Clinical Sciences, Agency for Science, Technology & Research, Singapore, Singapore

**Keywords:** Genomic analysis, Imaging, Genome informatics, Translational research, Genetics of the nervous system, Cardiovascular diseases, Psychiatric disorders

## Abstract

Cardiometabolic and psychiatric disorders often co-exist and share common early life risk factors, such as low birth weight. However, the biological pathways linking early adversity to adult cardiometabolic/psychiatric comorbidity remain unknown. Dopamine (DA) neurotransmission in the striatum is sensitive to early adversity and influences the development of both cardiometabolic and psychiatric diseases. Here we show that a co-expression based polygenic score (ePGS) reflecting individual variations in the expression of the striatal dopamine transporter gene (*SLC6A3*) network significantly interacts with birth weight to predict psychiatric and cardiometabolic comorbidities in both adults (UK Biobank, *N* = 225,972) and adolescents (ALSPAC, *N* = 1188). Decreased birth weight is associated with an increased risk for psychiatric and cardiometabolic comorbidities, but the effect is dependent on a striatal *SLC6A3* ePGS, that reflects individual variation in gene expression of genes coexpressed with the *SLC6A3* gene in the striatum. Neuroanatomical analyses revealed that SNPs from the striatum *SLC6A3* ePGS were significantly associated with prefrontal cortex gray matter density, suggesting a neuroanatomical basis for the link between early adversity and psychiatric and cardiometabolic comorbidity. Our study reveals that psychiatric and cardiometabolic diseases share common developmental pathways and underlying neurobiological mechanisms that includes dopamine signaling in the striatum.

**Supplementary Information:**

The online version contains supplementary material available at 10.1038/s41598-024-78465-5.

## Introduction

The co-occurrence of more than one chronic disease^1^ has high prevalence in primary care settings^2^, inflating health care utilization and functional disability^3^. Psychiatric and cardiometabolic disorders, which are highly comorbid^4,5^, rank amongst the leading global causes of disability-adjusted life years worldwide^6,7^. Prospective studies show a bi-directional relationship between psychiatric and cardiometabolic conditions^8^. Meta-analytic evidence from longitudinal studies indicates that diabetes increases the risk for depression by approximately 25% and that depression increases the risk for type 2 diabetes by 40–60%^7,9^. The odds for depression also increase with one or more non-psychiatric coexisting chronic conditions, especially coronary artery disease, chronic arthritis, and stroke^10^. Anxiety is also associated with 41% increased risk of developing cardiovascular disease^11^. Among adult patients with schizophrenia, the prevalence of diabetes averages 15%, which is higher than the 10% prevalence of diabetes in the general population. This association persists even after controlling for factors such as obesity and the use of antipsychotic drugs^12^.

The underlying mechanism for these comorbidities remains unknown, but an emerging explanation is that psychiatric and cardiometabolic disorders share common developmental pathways. For example, low birth weight, a prevalent form of early life adversity^13^, is specifically linked to cardiometabolic^14–18^ and psychiatric disorders^19–24^. One in four newborns worldwide are considered *small vulnerable newborns* experiencing significant intrauterine adversities, affecting their growth and development, with long-term consequences that reach the societal level independently of the economic status of the country^25^. There are many mechanisms involved with this condition, including genetic and epigenetic factors, maternal-fetal environment – such as mother’s age, ethnicity, and geographical location, maternal malnutrition, placental size and dysfunction, presence of maternal illness, and substance exposure (e.g., smoking, alcohol, drugs). Therefore, being born small is an insightful quantitative proxy for adversity exposure during pregnancy^26^ and associated with increased morbimortality throughout the life course^18^.

An obvious question concerns the biological mechanisms that underlie such a developmental trajectory involved in the development of cardiometabolic and psychiatric comorbidities. The brain dopamine system is highly sensitive to early adversity^27^ and proposed as a mechanism underlying developmental pathways to multiple psychiatric and metabolic comorbidities^28,29^. Early life adversity such as fetal growth restriction that leads to low birth weight alters dopaminergic signaling^30–32^. Dysfunction of dopamine neurotransmission in both the ventral and dorsal striatum associates with depression^33^, as well as dysregulated food intake and altered energy homeostasis^31,32,34^. Striatal dopamine signaling also appears to regulate systemic glucose metabolism in humans^35^. The striatum harbors dopaminergic neurons^36^ and the striatal dopamine transporter (DAT) is a critical regulator of striatal dopamine release and reuptake^37^. Dopamine signaling is influenced by core metabolic hormones such as leptin and insulin, through their actions on the expression and function of DAT^34,38^, which is encoded by the *SLC6A3* (solute carrier family 6 member 3) gene.

Based on the large evidence supporting the relation between metabolism, mental health and striatal dopaminergic neurotransmission, as well as the effects of early adversity on striatal dopamine function, we hypothesized that the striatal *SLC6A3* gene network underlies the association between early life adversity and the comorbidity between psychiatric and cardiometabolic disorders in humans. We therefore aimed to test if individual differences in the function of a striatal *SLC6A3* gene network might moderate the effects of early life adversity on psychiatric and cardiometabolic comorbidities in adults and adolescents. To achieve this, we created a *SLC6A3* striatal co-expression-based polygenic score (striatum *SLC6A3* ePGS) reflecting the genetic capacity for expression of the striatal DAT1 gene network (possibly influencing dopamine signaling) and analyzed the effect of its interaction with birth weight on the comorbidity of psychiatric and cardiometabolic conditions in adults (UK Biobank) and adolescents (Avon Longitudinal Study of Parents and Children, ALSPAC).

## Methods

### Participants

We used genomic and phenotypic data from two cohorts, one from adults (Uk Biobank), and one from adolescents (Avon Longitudinal Study on Parents and Children, ALSPAC).

#### Adult cohort

The UK Biobank is a large population-based study from the United Kingdom^39^. Participants, aged 37–73, were recruited between 2006 and 2010 resulting in 502,543 subjects. Detailed description of the inclusion/exclusion criteria for the current analysis and the corresponding sample size at each step can be found in *Supplementary information*, Supplementary Fig. 1. After all exclusion and inclusion criteria, the number of subjects that remained for the analysis was 225,972 (mean age = 55.22, SD = 8.08) (Table 1). We used all the data available for the brain imaging analysis considering the inclusion/exclusion criteria (*Supplementary information*, Supplementary Fig. 1, N = 11,167, mean age = 53.86, SD = 7.39).

**Table 1:** Description of the baseline characteristics in UK Biobank sample and associations with striatum *SLC6A3* ePGS.

UK Biobank (*n* = 225,972)				
Characteristics	Mean / N	SD/%	ePGS correlation/mean difference	p_value
Sex - Male	88,939	39.358%	0.007	0.109
Birth weight (grams)	3317.492	658.366	0.008	< 0.001
Completed full-time education at 14-years of age or younger	1667	1.159%	− 0.025	0.308
Age at recruitment (years)	55.218	8.079	0.013	< 0.001
Townsend deprivation index at recruitment	− 1.482	2.983	− 0.027	< 0.001
BMI at recruitment	27.265	4.866	− 0.001	0.570
ICD10 F10-F19 Mental and behavioural disorders due to psychoactive substance use	9278	4.106%	− 0.009	0.363
ICD10 F20-F29 Schizophrenia, schizotypal and delusional disorders	556	0.246%	− 0.022	0.613
ICD10 F30-F39 Mood [affective] disorders	8539	3.779%	0.008	0.469
ICD10 F40-F48 Neurotic, stress-related and somatoform disorders	5541	2.452%	− 0.015	0.272
ICD10 E11 Non-insulin-dependent diabetes	10,269	4.544%	− 0.037	< 0.001
ICD10 I70 Atherosclerosis	632	0.28%	0.001	0.978
ICD10 I63 Cerebral infarction	1758	0.778%	− 0.006	0.809
ICD10 I20-I25 Ischaemic heart diseases	15,389	6.81%	− 0.002	0.815

Townsend deprivation index in UK Biobank: A measure of the level of social deprivation that a person lives in. The index was calculated based on previous national census. Participant is given a score reflecting the output area in which their postal code is located. Four key aspects are considered in the index: the percentage of unemployment, overcrowded households, households without a car and non-home ownership.

#### Adolescent cohort

 To explore our findings in an earlier developmental time point we used data from the Avon Longitudinal Study of Parents and Children (ALSPAC) cohort^40–42^. This is a transgenerational prospective observational cohort that recruited 14, 541 pregnant women residents in Avon County, UK. Additional recruitment (*N* = 913) was performed later during Phases II, III and IV respectively, bringing the total sample size of prospective mother-child dyads to 15,658. For more information on ALSPAC variables, please see http://www.bristol.ac.uk/alspac/researchers/our-data/. Data from the adolescent offspring aged between 15.5 and 17.5 were used in this study. Only subjects with available phenotypic data of interest, early life adversity measure, in this case birth weight and genotyping data were considered for the analyses (*N* = 1,188) (Table 2). Detailed description of the inclusion/exclusion criteria and the corresponding sample size at each step can be found in *Supplementary information*, Supplementary Fig. 2.

**Table 2:** Description of the baseline characteristics in ALSPAC sample and associations with striatum *SLC6A3* ePGS.

ALSPAC (*n* = 1188)				
Characteristics	Mean / N	SD/%	ePGS correlation/mean difference	p_value
Sex - male	565	47.559%	0.086	0.144
Gestational Age (Weeks)	39.731	1.289	0.007	0.822
Birth weight (grams)	3508.933	450.425	0.034	0.239
SES (Crowding index above 1)	27	2.314%	0.080	0.665
Waist circumference (cm) at 15.5 years of age	76.237	8.262	0.028	0.329
SDQ Total difficulties score at 16.5 years of age	5.477	4.372	− 0.047	0.109
CIS-R Depression score at 17.5 years of age	0.283	0.747	0.014	0.640
CIS-R Anxiety score at 17.5 years of age	0.231	0.667	0.021	0.467
HOMA2-IR at 17.5 years of age	0.879	0.6	0.006	0.843
zBMI at 15.5 years of age	0.271	0.989	0.037	0.209

Low socioeconomic status (SES) in ALSPAC: crowding index higher than 0.75 at 2-year-and-9-month time point was considered as low SES. Crowding index was calculated by dividing the number of individuals living in the family dwelling by the number of rooms in the family dwelling and was used as a proxy measure of socioeconomic status.

See *Supplementary Information* Supplementary Methods for detailed description of the genotyping procedure for each cohort.

### Ethics approval and consent

#### UK Biobank

Informed consent was obtained from each participant, and the project has been approved by the North-West Multicentre Research 580 Ethics Committee (REC reference 11/NW/0382), the National Information Governance Board for Health and Social Care, and the Community Health Index Advisory Group for UK Biobank. Consenting participants provided baseline information, answered questions, had measurements and biological samples collected. This research has been conducted using the UK Biobank Resource under application number 41975.

#### ALSPAC

Participants provided written informed consent to participate in the study. Ethics approval for the study was obtained from the ALSPAC Ethics and Law Committee and the local research ethics committees (a full list of the ethics committees that approved different aspects of the ALSPAC studies is available at http://www.bristol.ac.uk/alspac/researchers/research-ethics/). Consent for biological samples has been collected in accordance with the Human Tissue Act (2004).

Consent for publication was obtained from UK Biobank and ALSPAC management teams. The use of these datasets was locally approved by the Centre intégré universitaire de santé et de services sociaux de l’Ouest-de-l’Île-de-Montréal Research Ethics Board under application number IUSMD-21-73.

### Identification of the striatal *SLC6A3* co-expression gene network and ePGS calculation

**Fig. 1:**
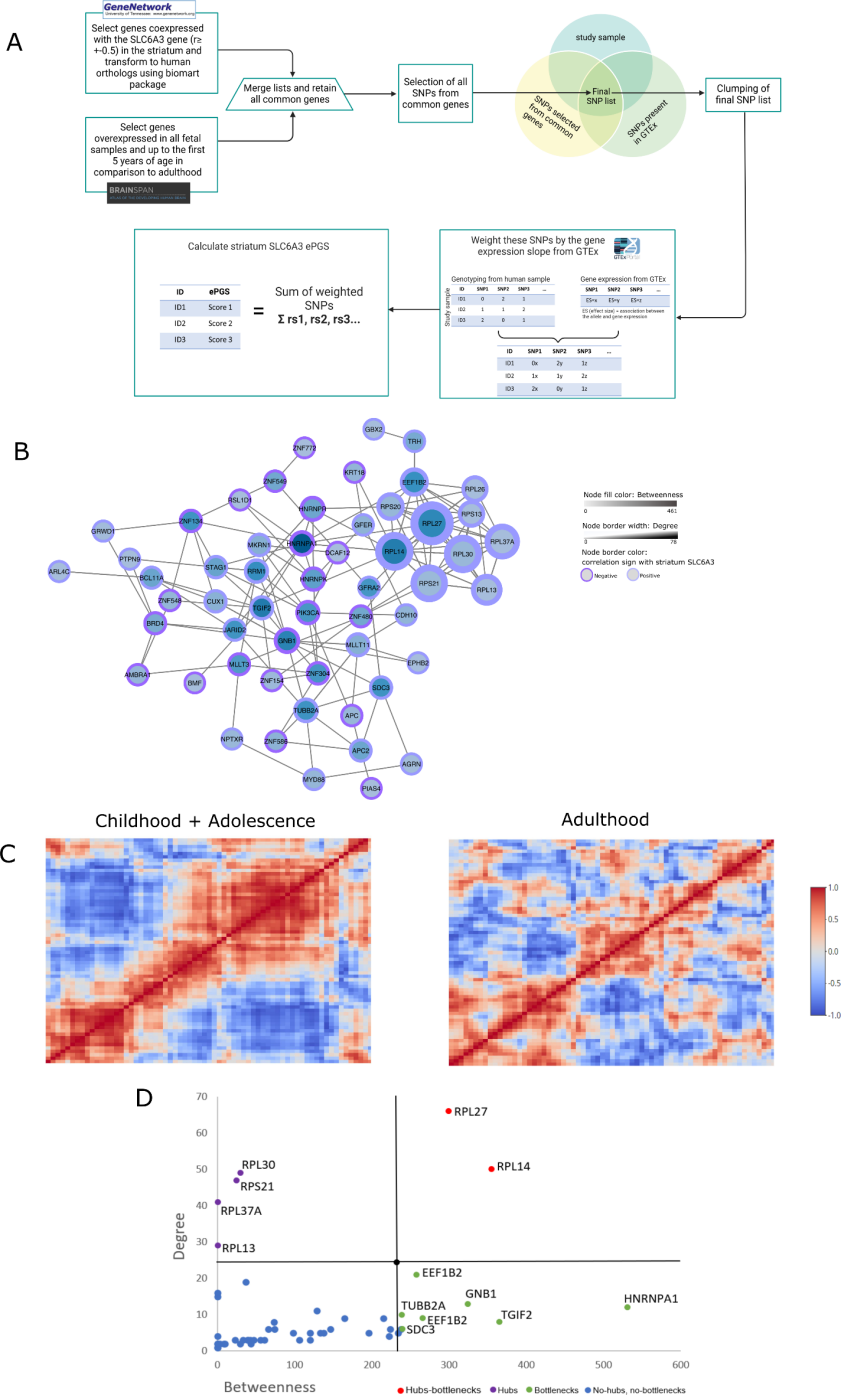
Construction and characterization of the striatum *SLC6A3* gene network. (**A**) calculation of the expression based polygenic score (ePGS) from the genes co-expressed with the *SLC6A3* gene in striatum. GeneNetwork was used to generate a list of genes co-expressed with *SLC6A3* in striatum in mice, which were then converted to human orthologs. BrainSpan was used to identify genes overexpressed within striatum in fetal samples and up to 5 years of age in comparison to adult samples. All SNPs from these genes, common between the study sample and GTEx databases, were retained and included in the final list of SNPs. This final list was subjected to linkage disequilibrium clumping, with removal of highly correlated SNPs. Next, for each SNP, a number of alleles at a given SNP from each participant’s genotype (rs1, rs2…) was multiplied by the estimated effect of the genotype-gene expression association from GTEx. The sum of these values over all SNPs provides the striatum *SLC6A3* ePGS. (**B**) striatum *SLC6A3* ePGS co-expression gene network. Co-expression pattern was mined from GeneMANIA^63^. The color of the node border represents the correlation sign with the *SLC6A3* gene according to GeneNetwork co-expression matrix (dark purple represents negative and light purple positive correlation). Node color intensity represents betweenness (number of times a node acts as a bridge between nodes). Node border width represents the number of connections a node has with other nodes (total degree). (**C**) Co-expression of genes included in the striatum *SLC6A3* gene network in humans at different ages according to BrainSpan. (**D**) Topological properties of the striatum *SLC6A3* gene network, showing hubs (with degrees higher than + 1SD above mean), bottlenecks (betweenness higher than + 1SD above the mean), and hub-bottlenecks. Lines in black indicate mean + 1 SD for degrees and betweenness. Hub and hub-bottleneck genes are related to ribosomal structure. Among the bottleneck genes, HNRNPA1 is involved in the packaging of pre-mRNA into particles and transport from the nucleus to the cytoplasm, as well as splicing. The SDC3 gene may play a role in cell shape organization and has been associated with obesity^77^.

Figure 1A shows the steps involved in the identification of the gene co-expression networks and the calculation of the ePGS score. The ePGS was calculated considering genes co-expressed with the *SLC6A3* gene in the striatum. As described previously^43–50^, we began by using brain region-specific RNA sequencing data from mice available at GeneNetwork (http://genenetwork.org/, HBP Rosen Striatum M430V2 (Apr05) RMA Clean)^51^ to identify *SLC6A3* co-expressed genes (absolute value of co-expression correlation with *SLC6A3* gene greater or equal to *r* = 0.5). GeneNetwork was used to obtain gene expression from rodents since our previous findings demonstrated multiple effects of early life adversities, especially poor fetal growth, on dopaminergic mesocorticolimbic system in rodents^30–32,52–56^. We then converted *SLC6A3* co-expressed genes to human orthologs by using the biomaRt package^57^. Since we were interested in gene networks that were active during the early developmental period in which adversity occurred and when the brain is still undergoing core maturational processes in humans, we used BrainSpan to select autosomal transcripts expressed at least 1.5-fold more during fetal and childhood periods (0–60 months after birth) in comparison to adulthood (20–40 years of age). This process resulted in a list of striatal *SLC6A3* co-expressed genes. We then mapped all the existent SNPs in the human ortholog genes comprising the striatum *SLC6A3* gene network using biomaRt package^57^ in R and gathered all gene-SNP pairs from the GTEx dataset in human striatum. These lists were merged with the genotyping data from UK Biobank and ALSPAC cohorts, respectively, retaining only common SNPs and subjecting the final SNP lists to linkage disequilibrium clumping (r2 < 0.2) within 500 kb radious to eliminate redundant SNPs. The process resulted in 1532 independent functional SNPs retained in UK Biobank and 1663 SNPs in ALSPAC. The final score included 67 genes in our discovery sample (UKB) (*Supplementary information*, Supplementary Table 1).

To calculate the striatal *SLC6A3* ePGS, the number of effect alleles (genotype information from the study samples) at a given SNP was weighted using the estimated brain-region-specific effect of the SNP on gene expression from the GTEx data^58^. We also accounted for the direction of the co-expression of each gene with *SLC6A3* in the network, by multiplying the weight by -1 in case the expression of a gene was negatively correlated with the expression of the *SLC6A3* gene in the network – therefore, the higher the score, the higher the expression of the genes that compose the network. The sum of the weighted values from all SNPs for each individual in the cohorts resulted in the region-specific striatal scores. The striatal *SLC6A3* expression-based polygenic score (ePGS) is a continuous measure that reflects variation of gene expression of the genes co-expressed with the *SLC6A3* gene in the striatum.

### Comparison between polygenic risk scores and ePGS

To compare the results obtained with the striatum *SLC6A3* ePGS, we generated traditional polygenic risk scores (PRS) using our accelerated pipeline (https://github.com/MeaneyLab/PRSoS)^59^. A traditional PRS is a cumulative score calculated based on a relevant GWAS that represents a genetic risk for a certain health outcome or trait^60^. The sum of the allele count weighted by the effect size across all SNPs in GWAS was used to calculate type 2 diabetes^61^ and major depression disorder^62^ PRSs. The number of SNPs included was defined based on the number of SNPs present in our striatum *SLC6A3* ePGS calculated in the discovery cohort. For MDD PRS we used the GWAS results that were obtained without UK Biobank or 23andMe subjects.

### Functional enrichment analysis

Enrichment analysis was performed using MetaCore^®^ software from Clarivate Analytics (https://portal.genego.com) to characterize the putative biological functions associated with the striatal *SLC6A3* co-expression gene network. Genes that comprise the striatal *SLC6A3* ePGS were used in the analysis and the whole genome was used as a background. The significance was considered for the false discovery rate (FDR) adjusted p-value < 0.05. To investigate network centrality measures, co-expression patterns were mined from geneMANIA^63^.The gene interactions were then visualized using the Cytoscape^®^ software^64^. The nodes are the elements of a network (genes) and edges are the connections between these elements. Bottleneck genes are defined as those having a high betweenness (the extent to which genes act as ‘bridges’ between other genes in a network), hub genes are defined as those with a high degree (genes with more connections to other genes). To analyze the topological properties associated with this gene network, the CentiScaPe app in Cytoscape^®^ was used to calculate the degree and betweenness of each gene. We used this information to define the “hub genes” within the network, characterized as nodes with degrees higher than + 1SD above the mean; and the “bottlenecks” characterized as nodes with betweenness higher than + 1SD above the mean. A gene that is both bottleneck and hub was considered as a central node of the network^65^. We also mined protein-protein (PPI) network interactions using the STRING database (https://string-db.org)^66^ and the striatum *SLC6A3* ePGS genes, with the objective to query the physical interactions of the genes that compose our genetic score. Although we mapped the mice co-expressed gene list to human orthologs, not necessarily the co-expression features would be recapitulated in humans. In order to confirm if the genes that comprised the striatal *SLC6A3* ePGS are co-expressed in humans and to analyze their patterns of co-expression during different life periods in humans, we used the gene expression data from human postmortem samples from the BrainSpan database^67^ (see *Supplementary information*, Supplementary Methods).

### Outcome measures

#### Adult cohort

Psychiatric disorder diagnosis was defined based on the primary or secondary diagnosis of a mental, mood, schizophrenia and neurotic disorders according to participants hospital inpatient records, coded according to the International Classification of Diseases version 10 (ICD-10)^68^ (UK Biobank field 41270; ICD10 codes: F10-F19 Mental and behavioural disorders due to psychoactive substance use, F20-F29 Schizophrenia, schizotypal and delusional disorders, F30-F39 Mood [affective] disorders, F40-F48 Neurotic, stress-related and somatoform disorders). Cardiometabolic disorders diagnosis was defined by the ICD-10 codes from chapter IV Endocrine, nutritional and metabolic diseases and chapter IX Diseases of the circulatory system (UK Biobank fields: 41270; ICD10 codes: E11-Non-insulin-dependent diabetes, I70-Atherosclerosis, I63-Cerebral infarction, I20-I25 Ischaemic heart diseases). The presence of at least one mental disorder diagnosis and at least one cardiometabolic diagnosis was considered a comorbidity case. Comorbidity variable was coded as a binary variable (1 = “yes” or 0 = “no”). T1 structural brain MRI pre-processed imaging data were generated by an image-processing pipeline developed and run on behalf of the UK Biobank^69^ (*Supplementary information*, Supplementary Methods).

#### Adolescent cohort

 No diagnoses for the psychiatric and cardiometabolic disorders noted above were available in ALSPAC. As recommended by the American Academy of Pediatrics (AAP)^70^, we defined disease risk in adolescents using continuous measures of Total difficulties score measured by the Strengths and Difficulties Questionnaire, depression and anxiety scores measured by Computerized Interview Schedule – Revised (CIS-R), Homeostatic Model Assessment of Insulin Resistance (HOMA-IR), and waist circumference (cm) (*Supplementary information*, Supplementary Methods, Supplementary Table 2, Supplementary Fig. 3). We then characterized two groups of children: low and high cardiometabolic and psychiatric comorbidity risk (see Statistical Analysis).

### Statistical analysis

Statistical analysis were performed using R^71^. For the descriptive statistics, the ePGS groups were defined by median split, and a comparison between low and high ePGS groups was done using Student t test for continuous variables and a chi-square test for categorical variables (Tables 1 and 2). Significance levels for all tests were set at *p* < 0.05.

We performed cluster analysis using the mclust package to construct the comorbidity risk variable in ALSPAC adolescent sample^72^. This algorithm applies a model-based classification and density estimation of the z-standardized variables based on finite Gaussian mixture modelling. The method assumes that predictors can be explained by an underlying latent categorical variable (cluster) that represents distinct profiles within the sample, both in a qualitative and quantitative manner. We defined a priori a cluster size solution of two (lower and higher risk for comorbidity). All predictors were z-transformed and adjusted for sex prior to entering the clustering procedure. Regression analysis was carried out to demonstrate the difference between the two clusters in the means for each variable used in the cluster analysis (*Supplementary information*, Supplementary Table 2). The resulting cluster membership, which represented comorbidity risk, was coded as a binary variable (1= “yes comorbidity” or 0= “no comorbidity”).

The gene by environment (G-E) interaction effect on binary outcomes was explored by logistic regression analysis. Birth weight as a continuous variable was used as a proxy for early life environment exposure in UK Biobank (variable ID20022) and ALSPAC. Early life adversity (E), striatal *SLC6A3* ePGS (G) and the interaction term between them were included in the model as main predictors for both cohorts. UK Biobank analyses were also adjusted by sex, age, the first forty genetic principal components, genotyping array, and assessment center, and ALSPAC analyses were adjusted by sex and the first ten genetic principal components. In case of a significant gene by environment (G-E) interaction effect, post hoc simple slope analysis was performed to investigate how the environment effect varies as a function of the genetic background^73^. The directionality of the G-E effect was explored in the UK Biobank, our discovery cohort, using a two-tailed P-value threshold. The directionality of the G-E effect on ALSPAC was anticipated based on the finding from UK Biobank, thus a one-tailed P-value threshold was considered.

The relation between early life adversity, ePGS and gray matter density in UK Biobank was analyzed in a multivariate parallel independent component analysis (pICA). This analysis was applied to identify the effect of early life adversity on the relation between two different data modalities (genetic and gray matter density) in a data-driven manner^74^. This analysis separately estimates the maximum independent components within each data modality while also maximizing the association between modalities using an entropy term based on information theory^74^. Each SNP that composes the striatal *SLC6A3* ePGS weighted by striatal GTEx data (genotype * GTEx striatum gene expression slope for each SNP) and whole brain voxel based gray matter density were used in the analysis. Weighted SNPs were adjusted for the genetic principal components (ancestry). The subjects were split in two groups according to the birth weight (low birth weight group: subjects with birth weight < = 2.5 kg, *n* = 953) and a randomly selected group of non-low birth weight individuals (subjects with birth weight > 2.5 kg, *n* = 953, please see https://www.who.int/data/nutrition/nlis/info/low-birth-weight), since there was a large discrepancy between cases and controls sample size within the subsample of individuals with T1 structural brain MRI available. Comparison between low birth weight and randomly selected non-low birth weight individuals on main descriptive variables can be seen on Supplementary information (*Supplementary information*, Supplementary Table 5). Comparison between the randomly selected group and the full sample of non-low birth weight individuals with MRI available can be seen on Supplementary information (*Supplementary information*, Supplementary Table 6). T1 structural brain MRI pre-processed images were adjusted by age and sex (See *Supplementary information*, Supplementary Methods). The Fusion ICA Toolbox (http://mialab.mrn.org/software/fit/) within MATLAB^®^ R2019 was used to run the analysis. The number of independent components was estimated using minimum description length criteria^74^ for the MRI modality and SNP dimensionality inside the toolbox for the genetic modality. Components for both modalities were converted to z-scores and a threshold at |Z| > 2.5 was used to identify significant brain regions and SNPs that contributed the most for the component overall pattern^74^. Loading coefficients, which describe the presence of the identified component across subjects^74^, were extracted for each component, modality, and subject. The mean subject-specific loading coefficients of these components from low birth weight and non-low birth weight groups were compared using Student’s t-test. Brodmann areas defined by talairach coordinates were used to identify the anatomical classification of brain areas included in the identified MRI component^75,76^. The significant SNPs (|Z| > 2.5) from the identified genetic component were analyzed using MetaCore^®^, to identify associated gene ontology processes terms (See Fig. 3A for graphical representation of pICA analysis).

## Results

### Characteristics of the striatal *SLC6A3* gene network

We developed a polygenic score to explore the genetic moderation of early life adversity on psychiatric and cardiometabolic comorbidities focusing on a specific gene network (Fig. 1A and B). We first used brain region-specific RNA sequencing data from mice available at GeneNetwork (http://genenetwork.org/)^51^ to identify genes co-expressed with the *SLC6A3* gene in the striatum. These genes were then converted to human orthologs (**Supplementary Tables 1** and Fig. 1B). This list was used to inform the calculation of the expression-based polygenic score (Striatum *SLC6A3* ePGS) in UK Biobank and ALSPAC participants as described in the Methods.

To investigate if mouse-generated *SLC6A3* gene network was co-expressed in humans, we queried the gene co-expression patterns of the striatum *SLC6A3* gene network throughout human development using gene expression data from human postmortem samples^67^. A high co-expression was expected in childhood/adolescence, as the striatum *SLC6A3* gene network was enriched for genes overexpressed in this period of life (see Fig. 1C and *Supplementary information*, Supplementary Methods). Prominent gene co-expression clusters were also seen in adults (Fig. 1C). These findings confirm that the striatal *SLC6A3* gene network, originally from murine data, is also co-expressed in humans, and that co-expression is observed at different ages. When visualizing and exploring the network properties (Fig. 1D), we observed that the central gene (hub) and the hub-bottleneck genes are related to ribosomal structure. Among the bottleneck genes, which are important connectors between groups of genes, we observed HNRNPA1, which is involved in the packaging of pre-mRNA into particles and transportation from the nucleus to the cytoplasm, as well as splicing. We also observed SDC3 gene, which plays a role in cell shape organization and has been associated with obesity^77^. Protein-protein interactions of the striatum *SLC6A3* co-expression network mined from STRING revealed that the network has significantly more interactions than expected by chance (*P* < 1.0e-16), suggesting that a significant number of the genes in this co-expression network also have physical interactions at the protein level.

The main gene ontology processes terms associated with the network (*Supplementary information*, Supplementary Figure S4) include: insulin signaling and response terms (Insulin receptor signaling pathway via phosphatidylinositol 3-kinase; Insulin receptor signaling pathway; Cellular response to insulin stimulus; Response to insulin), ribosome production related terms (Ribosome biogenesis; Ribosomal large subunit biogenesis), dopamine receptor signaling pathway (Adenylate cyclase-activating dopamine receptor signaling pathway) and inflammatory response related terms (Regulation of cytokine production involved in inflammatory response; Negative regulation of cytokine production involved in inflammatory response).

###  Striatum *SLC6A3* ePGS moderates the association between birth weight and the risk for psychiatric and cardiometabolic comorbidities in adults

Lower birth weight was associated with the presence of comorbidities in UK Biobank (b= -0.206, Odds ratio (OR) = 0.814, 95% confidence interval (95% CI): 0.781–0.847, *P* < 0.001). However, in ALSPAC this association was not significant (b= -0.091, OR = 0.913, 95% CI:0.680–1.228, *P* = 0.548).

**Fig. 2:**
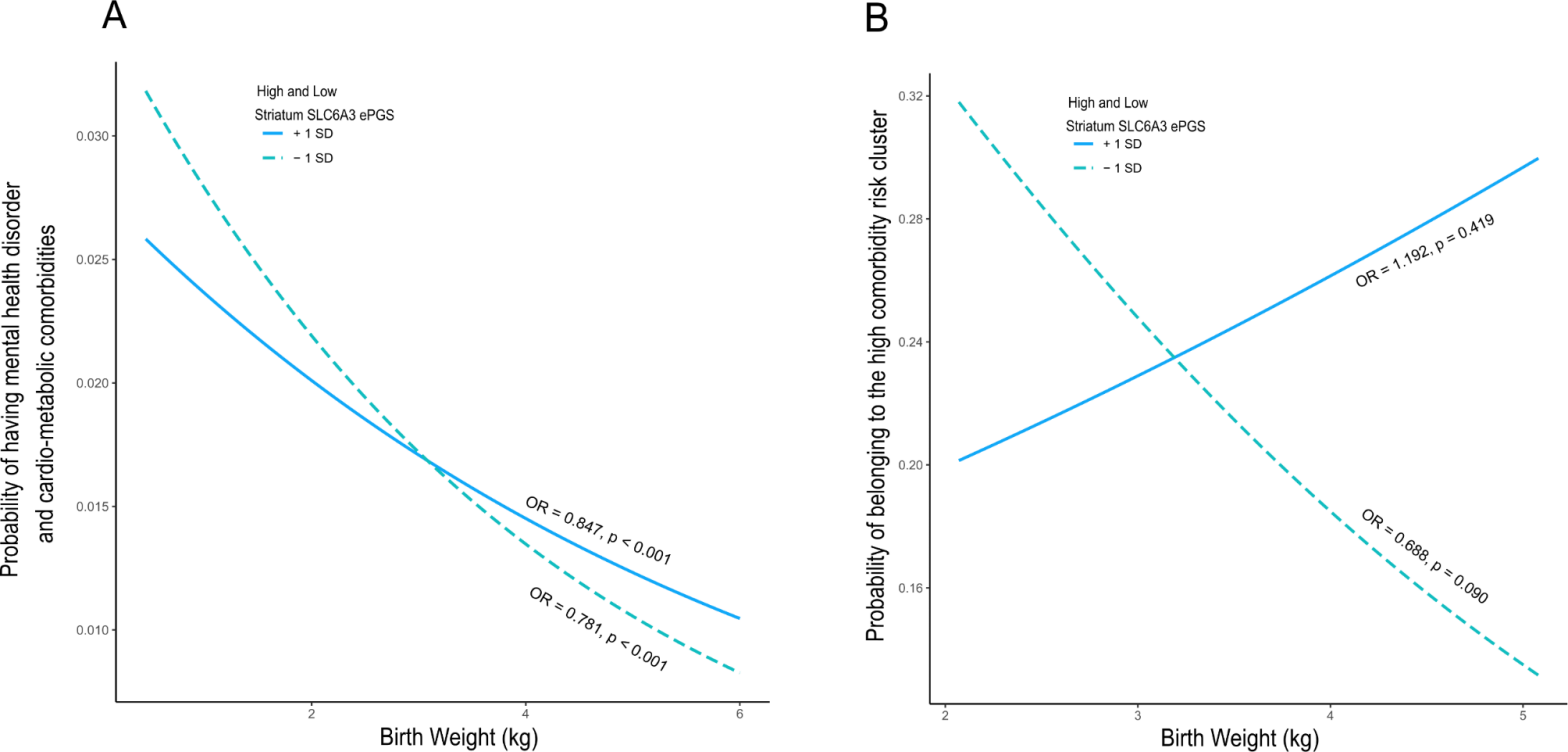
Striatum *SLC6A3* ePGS moderates the effect of early life adversity on the risk for mental health disorder and cardiometabolic comorbidity. Probability of having comorbidity in individuals with high and low striatum *SLC6A3* ePGS as a function of birth weight. A, UK Biobank cohort, *N* = 225,972. The risk for comorbidity increases as birth weight decreases, especially at lower ePGS values (Low ePGS: b = -0.247, OR = 0.781, *P* < 0.001, 95% CI 0.738–0.826; High ePGS: b = -0.166, OR = 0.847, *P* < 0.001, 95% CI 0.800–0.897). B, ALSPAC cohort, *N* = 1,188. The risk for comorbidity increases as birth weight decreases, especially at lower ePGS values (Low ePGS: b = -0.373, OR = 0.688, 95% CI 0.447–1.066, *P* = 0.090; High ePGS: b = 0.176, OR = 1.192, *P* = 0.419, 95% CI 0.779–1.825).

For the UK Biobank there was no significant main association of the ePGS with comorbidity (b = 0.006, OR = 1.006, 95% CI: 0.977–1.036, *P* = 0.678). In contrast, and consistent with our anticipated hypothesis, there was a significant interaction effect between the striatum *SLC6A3* ePGS and birth weight on the presence of psychiatric and cardiometabolic comorbidities in UK Biobank adults (b = 0.042, OR = 1.043, 95% CI: 1.001–1.086, *P* = 0.044). The risk for comorbidity increased as birth weight decreased, especially at lower ePGS values (Low ePGS: b = -0.247, OR = 0.781, *P* < 0.001, 95% CI 0.738–0.826; High ePGS: b = -0.166, OR = 0.847, *P* < 0.001, 95% CI 0.800–0.897). (Fig. 2A). (As we considered birth weight as a continuous variable ranging from low to high values and comorbidity as a dichotomous variable, with the presence of comorbidity computed as 1 and absence as 0, the odds ratio represents the negative association between birth weight and the probability of having comorbidity. Results are presented from the perspective of low birth weight).

In ALSPAC adolescents the G-E model revealed a significant interaction effect between the striatum *SLC6A3* ePGS and birth weight on the probability of belonging to the high comorbidity risk cluster (b = 0.271, OR = 1.311, 95% CI: 1.015 – Inf, *P* = 0.041, *n* = 1,188). The risk for of belonging to the high comorbidity risk cluster increased as birth weight decreased, especially at lower ePGS values (Low ePGS: b = -0.373, OR = 0.688, 95% CI 0.447–1.066, *P* = 0.090; High ePGS: b = 0.176, OR = 1.192, *P* = 0.419, 95% CI 0.779–1.825). (Fig. 2B). Similar to the findings in adults, there was no significant main effect association of the striatum *SLC6A3* ePGS on the comorbidity risk (b = 0.086, OR = 1.090, 95% CI: 0.957–1.240, *P* = 0.195). These results indicate a developmental trajectory, in which early indicators of risk to develop psychiatric and metabolic comorbidities in adulthood can be seen in adolescents as a function of the interaction of the striatum *SLC6A3* co-expression gene network and birth weight.

To benchmark our method against the classical polygenic risk score derived from a GWAS, we performed the same G-E interaction analysis using birth weight and PRSs based on GWAS for major depressive disorder^62^ and type 2 diabetes^61^. These are phenotypes related to our main outcome, psychiatric and cardiometabolic comorbidity. We found significant main effects of Type 2 diabetes and MDD PRSs on the comorbidity outcome in UK Biobank, but not in ALSPAC (*Supplementary information*, Supplementary Table 3) and no significant G-E interaction on comorbidity using these PRSs in the UK Biobank or ALSPAC (*Supplementary information*, Supplementary Table 4).

### SNPs from the striatum *SLC6A3* ePGS are related to gray matter variations in the frontal cortex

**Fig. 3:**
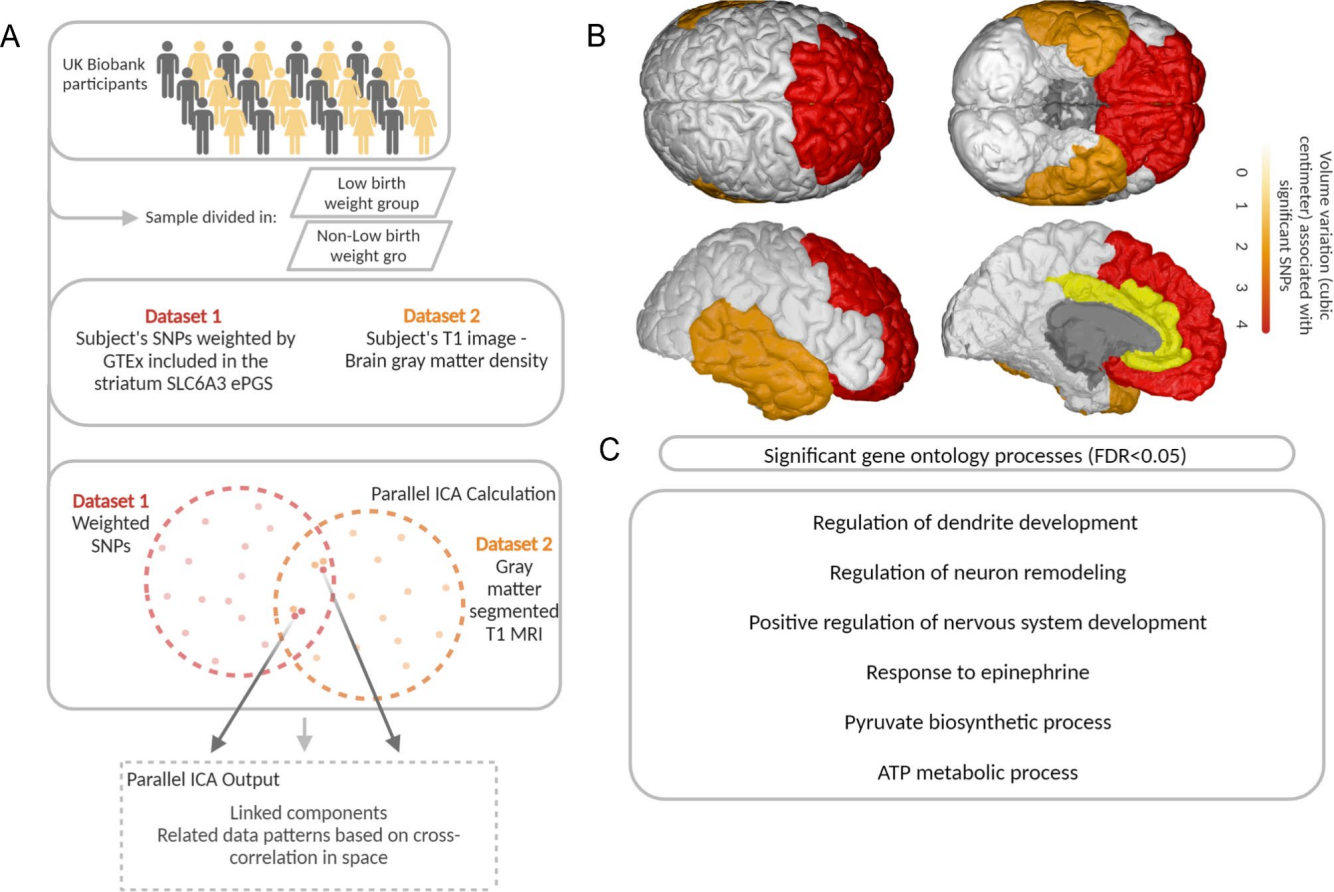
Parallel ICA analysis. (**A**) schematic representation of parallel ICA method. Two different data modalities (SNPs and voxel-based gray matter) were used to establish anatomical-functional correlations between the striatum *SLC6A3* ePGS and brain features from UK Biobank participants (*N* = 11,167). Participants were separated into low birth weight and normal birth weight groups. The analysis estimates the maximum independent components within each data modality separately while also maximizing the association between modalities using an entropy term based on information theory. (**B**) significant brain regions associated with SNPs from the striatum *SLC6A3* were frontal cortex, including the orbitofrontal and prefrontal cortex, cingulate cortex and temporal cortex. Color scheme represents the amount of volume variation (cubic centimeter) significantly associated with the subset of SNPs. (**C**) summary of significant gene ontology processes related to SNPs from the striatum *SLC6A3* ePGS associated with gray matter. Image created with BioRender.com (BioRender.com/f03l258) and open-source tool BrainPainter.

We then explored the neuroanatomo-functional relevance of the relation between the striatal *SLC6A3* gene network and early adversity. Functional refers to the variation in gene expression represented by the weight attributed to the SNPs that compose the striatum *SLC6A3* ePGS. We used a multivariate parallel independent component analysis (pICA)^74^ (Fig. 3A and *Supplementary information*, Supplementary Methods) and investigated correlations between the SNPs from the striatum *SLC6A3* ePGS and voxel-based gray matter density in UK Biobank participants from low birth weight and non-low birth weight groups. This analysis identifies independent components within each data modality separately (SNPs and MRI) while also maximizing the association between these two modalities. The estimated number of components for the MRI modality was 28 and for the genetic modality was 34. Only the most significantly linked pair of components that resulted from the multivariate analysis with higher correlation index value was selected to be further explored: the pair combining the genetic component 13 and MRI component 18 (*r*=-0.201, *p* = 6.779e^− 19^). A statistically significant difference between birth weight groups was observed for both the genetic component 13 (t = 2,214, *p* = 0.026) as well as the MRI component 18 (t=-3,318, *p* < 0.001). These differences between the adversity groups suggest that the relations between data pattern variations (i.e., the relationships between SNPs and gray matter) within this pair of components are significantly different between the two birth weight groups. We then explored the content of genetic component 13 and MRI component 18. The subset of significant SNPs within component 13 is related to variations in gray matter density in the frontal cortex, including the prefrontal cortex, and also more specifically the orbitofrontal cortex, part of the prefrontal cortex, cingulate cortex and temporal cortex (Fig. 3B). Enrichment analysis of this subset of significant SNPs (*Supplementary information* Supplementary Table 7) using MetaCore^®^ (FDR < 0.05) showed that the most significant gene ontology enrichment terms are related to regulation of dendrite development, regulation of neuron remodeling, positive regulation of nervous system development, pyruvate biosynthetic process, ATP metabolic process and response to epinephrine (Fig. 3C).

## Discussion

### Overview and discussion of main results

Our study suggests that being born with lower birth weight increases the risk for later comorbidities between cardiometabolic and psychiatric conditions in adulthood. In fact, being born at low birth weight, which reflects prenatal adversity^13^, independently associates with increased risk for developing both cardiometabolic^14–17^ and psychiatric disorders^19–24^ corroborating our findings. Our functional genomics approach provides evidence for the striatal *SLC6A3* co-expression gene network as a salient mechanism moderating this association. This finding is aligned with the critical role of the dopaminergic system in environmental responsivity^44,78^.

Although lower birth weight is associated with an increased risk for co-morbidity in both ePGS groups in the UK Biobank, low ePGS participants have significantly more risk than high ePGS individuals. In the low ePGS group in adolescence, there is a suggestion of increased risk of being part of a high comorbidity risk as birth weight decreases (*p* = 0.09). Although the simple slope for the high ePGS group in adolescence shows a positive inclination between birth weight and risk for comorbidity, this slope is not significant therefore the risk for comorbidity does not vary according to birth weight in the high ePGS group. No information on gestational age was available in the UK Biobank cohort. To maintain consistency, birth weight as a continuous variable and not corrected for gestational age was used in both cohorts. The lack of information about gestational age in our study may be especially affecting the adolescent analysis and this may potentially explain why the simple slope for the low ePGS group does not reach statistical significance, although the interaction between ePGS and birth weight is statistically significant in ALSPAC.

### Comparison between ePGS and PRS scores on main hypothesis testing

Based on the comparisons observed in this study, only the striatum *SLC6A3* ePGS was capable of capturing gene by environment interaction effects, while different PRSs did not significantly interact with early life adversity to predict the main outcome. GWAS-derived PRS reflect main genetic effects and thus are unlikely to capture individual differences in response to environmental variation. Indeed we found significant main effects of the PRSs of Major depressive disorder^62^ and Type 2 diabetes^61^ on comorbidity in UK Biobank. Overall, these results align with the well-known capacity of PRS to detect main genetic effects, as well as demonstrate the ability of our ePGS technique in identifying responsivity to environmental change as compared to traditional GWAS-based PRS^79^. PRS main effects were not observed in adolescents from ALSPAC probably due to the specificity of the GWAS to the features of the original discovery sample; the majority of GWASs are generated based on adult samples^80^, thus limiting the extrapolation of the effects in different ages.

### Discussion of enrichment analysis results

Our enrichment analysis showed that the striatal *SLC6A3* gene network is co-expressed in humans across childhood/adolescence and adulthood (Fig. 1C), which is aligned with our interaction between striatum *SLC6A3* ePGS and birth weight been observed both in adolescents and in adults. Our results therefore demonstrate that striatum *SLC6A3* ePGS is able to detect individual differences in response to early adversity at both ages.

We also identified putative biological mechanisms underlying the moderating effect of the striatum *SLC6A3* ePGS on the association between early adversity and cardiometabolic – psychiatric comorbidities (Fig. 1D and Supplementary Fig. 4). Central genes of the striatum *SLC6A3* network are related to ribosomal structure and the entire gene network is significantly enriched for ribosome production related terms (Ribosome biogenesis; Ribosomal large subunit biogenesis) as seen in our gene-ontology analysis. Our *SLC6A3* gene network is significantly enriched for inflammatory response related terms, especially cytokine production and not surprisingly, for dopamine receptor signaling pathway. (see *Supplementary information* Supplementary Discussion for complement discussion of enrichment terms related to ribosome functioning, inflammation and dopamine function)

Our striatum *SLC6A3* gene network is significantly enriched for insulin signaling and response related terms. Being born with low birth weight is associated with insulin resistance in children and adolescents^81^ and insulin resistance is a risk factor for cardiometabolic and brain-based disorders, including type II diabetes, cardiovascular disease, Alzheimer disease, and major depressive disorder^82,83^. Metformin, a medication to treat insulin resistance, has shown beneficial psychotropic effects in psychiatric conditions^84,85^. Evidence shows that insulin has a role in modulating mesocorticolimbic DA neurotransmission through different mechanisms, one of which is increasing DA reuptake by activating the phosphatidylinositol (PI) 3-kinase^38,86^. Insulin also reduces DA release in rodent nucleus accumbens and medial prefrontal cortex slices^87^. Our significant gene by environment results, using birth weight as our environmental proxy, corroborate with the literature showing elevated risk for developing psychiatric and cardiometabolic disorders among individuals born with low birth weight. Our genetic enrichment analysis results indicate that insulin signaling disturbances may be a potential mechanism involved in the interaction effect between birth weight and the striatum *SLC6A3* co-expression gene network on the risk for cardiometabolic and psychiatric comorbidities. This is aligned with many other studies suggesting that altered insulin function is an important mechanism linking early adversity to later disease^88,89^.

### Discussion of genomic-anatomical association results

The frontal, prefrontal and orbitofrontal cortices were related to the significant subset of SNPs identified by the pICA analysis (see *Supplementary information* Supplementary Discussion for complement discussion of the associated processes related to the subset of SNPs identified by the pICA analysis). This is aligned with evidence demonstrating that resting state functional connectivity between the orbitofrontal cortex and dorsolateral prefrontal cortex is altered in human individuals born small for gestational age, at different ages during development^90^.

The cingulate and temporal cortices also emerged as a significant brain regions in the pICA analysis. The anterior cingulate has been implicated in affective abnormalities in mood disorders and volume reduction in patients with major depressive disorder^91^. Abnormal posterior cingulate functional connectivity has also been reported in major depression^92^. Temporal lobe alterations are related to insulin resistance pathophysiology in different imaging modalities^93^. Interestingly, the relationship between genetic and MRI components was significantly different between the two early life adversity groups, suggesting that the biological mechanisms represented by the genetic component and the brain regions highlighted by the MRI component are relevant for the effects of early adversity on adult disease.

### Conclusion of potential biological mechanisms and anatomical regions involved in main results

Taken together, the evidence suggests that ribosomal function, inflammation, and insulin modulation of dopamine function may be underlying mechanisms by which the striatum *SLC6A3* gene network moderates the risk for developing psychiatric and cardiometabolic comorbidities in response to early life adversity. These mechanisms might be especially important in brain areas involving the prefrontal and orbitofrontal cortices, cingulate and temporal cortices.

### Limitations

Our study is limited by the fact that ePGS does not consider intronic regions, potentially ignoring other important regulatory elements. The complex data analysis presented on this study relies on a series of steps, which depend on a prior set of assumptions and the results presented here, based on secondary analysis, require validation by a longitudinal study. Our outcome measures related to psychiatric disorders in the UK Biobank are based on ICD-10 classifications derived from hospital inpatient data, not always attributed by mental health professionals or from structured clinical interviews which are considered the gold standard in psychiatric diagnosis. Similarly, in the ALSPAC cohort, indicators of risk for developing psychiatric disorders are collected through self-report measures. This situation is common in large nonspecialized data sources^94^, although it presents a limitation compared to cardiometabolic disorder diagnoses, which rely on objective biological measures, resulting in greater diagnostic accuracy. Although low birth weight is itself considered a form of early life adversity that impacts growth and development with long-term consequences^25^, the datasets used in this study provide limited information on possible causal prenatal adversity factors (e.g. maternal smoking or illness during gestation). Moreover, potential interactions between prenatal and postnatal stressors were not assessed. The pICA results are potentially limited by the diminished capacity of the technique in detecting subcortical regions, as the atlas used emphasizes cortical areas^95^.

## Conclusion

In sum, we observed that the association between environmental and genetic factors can place individuals at risk for adult comorbid chronic conditions from an early age, and that a striatal dopamine transporter gene network expression has a central role in moderating the association of the early environment with the risk for these diseases. These findings open opportunities for the exploration of the understudied field of precision prevention in pediatrics, and the potential design of more effective interventions and primary care strategies.

## Electronic supplementary material

Below is the link to the electronic supplementary material.

Supplementary Material 1

## Data Availability

The raw genetic and phenotypic data that support the findings of this study are available from UK Biobank (https://www.ukbiobank.ac.uk/) and ALSPAC (https://www.bristol.ac.uk/alspac/) but restrictions apply to the availability of these data, which were used under license for the current study, and so are not publicly available. Codes, variables processing information, and more details can be obtained by contacting the corresponding author at patricia.silveira@mcgill.ca.
