# Supplementary material for: Striatal dopamine gene network moderates the effect of early adversity on the risk for adult psychiatric and cardiometabolic comorbidity
Source: Sci Rep. 2024 Nov 9;14:27349. doi: 10.1038/s41598-024-78465-5 (PMC11550826; doi:10.1038/s41598-024-78465-5)
Supplement: Supplementary file 1 — Supplementary Material 1 [file 41598_2024_78465_MOESM1_ESM.pdf]

Supplementary Information

Striatal dopamine gene network moderates the effect of early adversity on the risk for adult psychiatric and cardiometabolic comorbidity

Supplementary Methods

Participants

UK Biobank – Adult cohort: UK Biobank contains information on participants' lifestyle and health data at baseline or follow-up, which were collected through questionnaires, physical measurements, and biological samples. For the purpose of this project, only unrelated subjects were considered in the analysis. Exclusion criteria were 1) participants who withdrew their consent from the study, 2) no genotyping data, 3) related participants (genetic kinship to other participants > 0.04), 4) inconsistencies in genetic and reported sex and 5) outliers for heterozygosity. All subjects selected for the analysis satisfied the following criteria: (1) have genotyping data available and (2) have both the diagnosis outcome and birth weight available. Detailed description of sample selection process can be found in **Supplementary Figure 1**.

ALSPAC – Adolescent cohort: Data were collected during clinic visits or with postal questionnaires. Please note that the study website contains details of all the data that is available through a fully searchable data dictionary and variable search tool at http://www.bristol.ac.uk/alspac/researchers/our-data/. The following inclusion criteria were applied: unrelated individuals, gestational age between 37 and 42 weeks inclusively, maternal age at delivery >= 18 years old, birth weight of at least 2000 grams, singleton pregnancies. Detailed description of sample selection process can be found in **Supplementary Figure 2**. The participant attrition block scheme differs slightly in originally projected ALSPAC participant numbers as we are using an earlier data file from 2019 to complete these analyses.

Genotyping

UK Biobank – Adult cohort: Blood samples from the UK Biobank were genotyped at the Affymetrix Research Services Laboratory in Santa Clara, California, USA. Genotyping was conducted using a bespoke BiLEVE Axiom array for 50,000 participants and the remaining 450,000 participants were genotyped using the Affymetrix UK Biobank Axiom array. The two SNP arrays are very similar with over 95% common marker content. Axiom Array plates were processed on the Affymetrix GeneTitan® Multi-Channel (MC) Instrument. Genotypes were then called from the resulting intensities in batches of ~4,700 samples (~4,800 including the controls) using the Affymetrix Power Tools software and the Affymetrix Best Practices Workflow. Individuals with the same genotype at any given SNP will cluster together in a two-dimensional intensity space (one dimension for each targeted allele). For the interim data release, Affymetrix performed further rounds of genotype calling using algorithms customized for the UK Biobank project. These algorithms targeted very rare SNPs with 6 or fewer minor alleles in a batch, and a subset of SNPs for which the generic calling algorithm did not perform optimally. After genotype calling, Affymetrix performed quality control in each batch separately, to exclude SNPs with poor cluster properties. If a SNP did not meet the

Affymetrix prescribed QC thresholds in a given batch, it was set to missing in all individuals from that batch. Hardy-Weinberg equilibrium was performed for each batch. Affymetrix also checked sample quality (such as DNA concentration) and genotype calls were provided only for samples with sufficient DNA metrics. For SNP-based QC metrics, only individuals with similar ancestry and the population structure were characterized by computing principal components using only UK Biobank individuals. The array also includes coding variants across a range of minor allele frequencies (MAFs), including rare markers (<1% MAF); and markers that provide good genome-wide coverage for imputation in European populations in the common (>5%) and low frequency (1–5%) MAF ranges. More information about the genotyping protocol, QC and imputation could be found in [1]. The population structure of the UK Biobank cohort was evaluated using fastPCA algorithm for principal component analysis[2]. To account for population stratification, the first forty principal components were included in the UK Biobank analysis.

ALSPAC – Adolescent cohort: Subjects in ALSPAC cohort were genotyped using the Illumina HumanHap550 quad genome-wide SNP genotyping platform by the Welcome Trust Sanger Institute (Cambridge, UK) and the Laboratory Corporation of America (Burlington, NC, US)[3]. The following quality control procedure was applied: participants with inconsistencies in self-reported and genotyped sex, minimal or extreme heterozygosity, high levels of individual missingness (>3%), and insufficient sample replication (IBD < 0.8) were excluded. SNPs with MAF <1%, call rate <95%, or those not in HWE ($p < 5 \times 10^{-7}$) were removed. Imputation was conducted using Impute v3 and Haplotype Reference Consortium (HRC) imputation reference panel (release 1.1). The resulting data set consisted of 8,365 individuals and 38,898,739 SNPs available for analysis. The population structure of the ALSPAC cohort was described using principal component analysis[4,5], which was conducted on the genotyped autosomal SNPs with MAF > 5% with the following pruning parameters for linkage disequilibrium: 100-SNP sliding window, an increment of 5 SNPs, and variance inflation factor threshold of 1.01. To account for population stratification, the first ten PCs were included in the analysis. Processing of the genotyping data was done using PLINK 1.9 [6](authors: Shaun Purcell, Christopher Chang; www.cog-genomics.org/plink/1.9/).

Gene expression levels at different developmental stages

In order to confirm if the genes that composed the striatum SLC6A3 ePGS are co-expressed in humans and investigate their patterns of gene co-expression during different life periods, we used the human postmortem striatal gene expression data from the BrainSpan database[7]. The co-expression patterns were analyzed during two different stages of development: childhood/adolescence (0 to 19 years old, N=7) and adulthood (20 to 40 years old, N=6). The analyses were carried out in R (https://www.r-project.org)[8] using the heatmaply package[9].

Outcome measures

UK Biobank – Adult cohort: To access the presence of psychiatric and cardio-metabolic disorders within the UK Biobank cohort, we utilized diagnostic terms from across all participants hospital inpatient records, coded according to the International Classification

of Diseases version 10 (ICD-10)[10]. The presence of at least one cardio-metabolic diagnosis and at least one psychiatric diagnosis was considered a comorbidity case.

Psychiatric disorder diagnosis definition:

Cases:

From Hospital Episodes Data from UK bodies (English HES Data, Scottish Morbidity Register, Patient Episode Data) (Fields 41270):
Any primary or secondary diagnosis of ICD-10 Codes for:
o ICD10 F10-F19 Mental and behavioural disorders due to psychoactive substance use'
o ICD10 F20-F29 Schizophrenia, schizotypal and delusional disorders
o ICD10 F30-F39 Mood [affective] disorders
o ICD10 F40-F48 Neurotic, stress-related and somatoform disorders

Controls:

No primary or secondary diagnosis of ICD-10 codes for:
o ICD10 F10-F19 Mental and behavioural disorders due to psychoactive substance use'
o ICD10 F20-F29 Schizophrenia, schizotypal and delusional disorders
o ICD10 F30-F39 Mood [affective] disorders
o ICD10 F40-F48 Neurotic, stress-related and somatoform disorders

Cardio-metabolic disorder diagnosis definition:

Cases:

From Hospital Episodes Data from UK bodies (English HES Data, Scottish Morbidity Register, Patient Episode Data) (Fields 41270):
Any primary or secondary diagnosis of ICD-10 Codes for:
o ICD10 E11 Non-insulin-dependent diabetes
o ICD10 I70 Atherosclerosis
o ICD10 I63 Cerebral infarction
o ICD10 I20-I25 Ischaemic heart diseases

Controls:

No primary or secondary diagnosis of ICD-10 codes for:
o ICD10 E11 Non-insulin-dependent diabetes
o ICD10 I70 Atherosclerosis
o ICD10 I63 Cerebral infarction
o ICD10 I20-I25 Ischaemic heart diseases

ALSPAC – Adolescent cohort: The indicators of risk to develop psychiatric disorders comprised: a) total difficulties score as measured by the Strengths and Difficulties Questionnaire[11] filled out by primary caregivers of 16.6 year old participants. This instrument evaluates behavioral problems, and a total difficulties score is computed by adding four domains of the scale (emotional symptoms, conduct problems, hyperactivity/inattention and peer relationship problems) that represent negative behaviors; b) depression score and c) anxiety score as measured by the Computerized Interview Schedule – Revised (CIS-R) that establishes the nature and severity of neurotic

symptoms[12], applied to 17.5 year old participants. The indicators of risk to develop metabolic disorders involved: d) insulin resistance as measured by the Homeostatic Model Assessment of Insulin Resistance (HOMA-IR), calculated using plasma fasting glucose (mmol/l) and insulin (pmol/l) levels collected at 17.5 years of age. The calculation followed the updated version of the HOMA-IR index developed by Wallace et al (2004)[13] and was computed using the HOMA2 calculation tool (http://www.dtu.ox.ac.uk/homacalculator/); and e) waist circumference (cm) measured at 15.5 years of age. Similar to UK Biobank, we created comorbidity risk variable based on the indicators of risk to develop psychiatric and metabolic disorders. Precisely, to construct comorbidity risk variable we performed cluster analysis on five risk indicators: Total difficulties, depression and anxiety scores, HOMA-IR and waist circumference. All predictors were z-transformed prior to entering the clustering procedure and adjusted by sex. We defined a cluster solution of two clusters, representing lower and higher risk for comorbidity. Regression analysis was carried out to demonstrate the difference between the means for each variable included in the cluster analysis (*Supplementary information*, **Supplementary Table 2, Supplementary Figure 3**).

Gray matter density in UK Biobank participants: T1 structural brain MRI pre-processed imaging data were generated by an image-processing pipeline developed and run on behalf of the UK Biobank[14]. High-resolution T1-structural images for the whole brain were acquired with straight sagittal orientation using a Siemens Skyra 3T running VD13A SP4, with a standard Siemens 32-channel RF receive head coil. The following parameters were used: resolution 1x1x1mm; field-of-view 208x256x256 matrix; 5 minutes duration; 1 mm isotropic resolution using 3D MPRAGE acquisition; in-plane acceleration iPAT=2; prescan-normalize. Full 3D gradient distortion correction (GDC) was applied to the original T1 image and the field of view (FOV) was cut down to reduce the amount of non-brain tissue. Tools used to achieve this include BET (Brain Extraction Tool), FLIRT (FMRIB's Linear Image Registration Tool), and the MNI152 "nonlinear 6th generation" standard-space T1 template. A non-linear registration to MNI152 space was used with FNIRT (FMRIB's Nonlinear Image Registration Tool). Using the inverse of the MNI152 alignment warp, a standard-space brain mask was transformed into the native T1 space and applied to the T1 image to generate a brain-extracted T1. Tissue-type segmentation was applied to T1 weighted images using FSL/FAST (FMRIB's Automated Segmentation Tool). Then, the T1-weighted gray matter images were selected and adjusted for age and sex. For each voxel separately, we applied linear regression analysis to regress the intensity on age and sex and used the residuals in p-ICA analysis.

Supplementary Discussion

Ribosomes functioning is highly related to cell growth and proliferation and protein synthesis in all cells. The ribosome biogenesis is a critical process to form mature ribosomes[15]. Dysfunction of ribosomal gene expression has been seen in animals' models of depression[16] and the use of anti-ribosomal P antibodies, that targets phosphorylated protein (P) components of ribosomes, was able to induce depression like behavior in mice[17]. Interestingly, the same antibody is used to detect systemic lupus erythematosus in humans[17]. This autoimmune disease is related to high levels of systemic inflammation. One of the central genes of our network, *GNB1*, has been implicated in regulation of

inflammasomes, multiple proteins complexes responsible for activation of inflammatory responses[18]. In fact, many patients with major depressive disorder have elevated levels of inflammatory cytokines [19] and there is evidence linking cardiometabolic syndrome to higher levels of circulating cytokines[20].

HNRNPA1, a central node of our network, is involved in mRNA transport and synthesis[21]. mRNA axonal transport and protein synthesis at the terminal is an important mechanism for regulation of neurotransmitter synthesis and reuptake[22]. Although *SLC6A3* gene expression occurs at the level of the ventral tegmental area, having *HNRNPA1* as a central node of our network could explain the presence of *SLC6A3* mRNA at the striatal terminal, consistent with numerous human post-mortem studies[23-26]. *SLC6A3* mRNA transport to terminals may be a key mechanistic feature of our *SLC6A3* striatum gene network. It has been shown that *SLC6A3* protein vesicular traffic has a limited contribution to *SLC6A3* concentration in synapses[27], hence other forms of regulation of *SLC6A3* availability in terminals – for instance via mRNA axonal transport and terminal protein synthesis – are likely in place, in agreement to our findings.

Another important gene in the network is the SDC3, that may play a role in cell shape organization and has been associated with obesity[28]. Obesity is related to increased risk for cardiovascular disease[29], high levels of inflammation and insulin resistance[30].

The subset of SNPs from the striatum *SLC6A3* ePGS that is related to gray matter density variations in our neuroanatomical-functional correlation analysis is associated with regulation of dendrite morphogenesis, neuron remodeling and positive regulation of nervous system development. These are important processes linked to the prolonged maturation of the mesocorticolimbic dopamine system during the life-course[31], which makes striatal dopaminergic axons especially vulnerable to environmental effects during development[32]. According to these findings, we recently showed that both rodent poor fetal growth and insulin treatment affect the expression of the Netrin-1/DCC axonal guidance cue system, which is involved in the maturation of the mesocorticolimbic DA circuitry[33] . Pyruvate biosynthetic process and ATP metabolic process also emerged as significant enrichment terms for the subset of significant SNPs related to gray matter density. Both processes have connections with insulin secretion: regulation of insulin secretion in pancreatic β cells is modulated by ATP synthesis and release in mitochondria[34] and by pyruvate transport through mitochondrial pyruvate carriers[35].

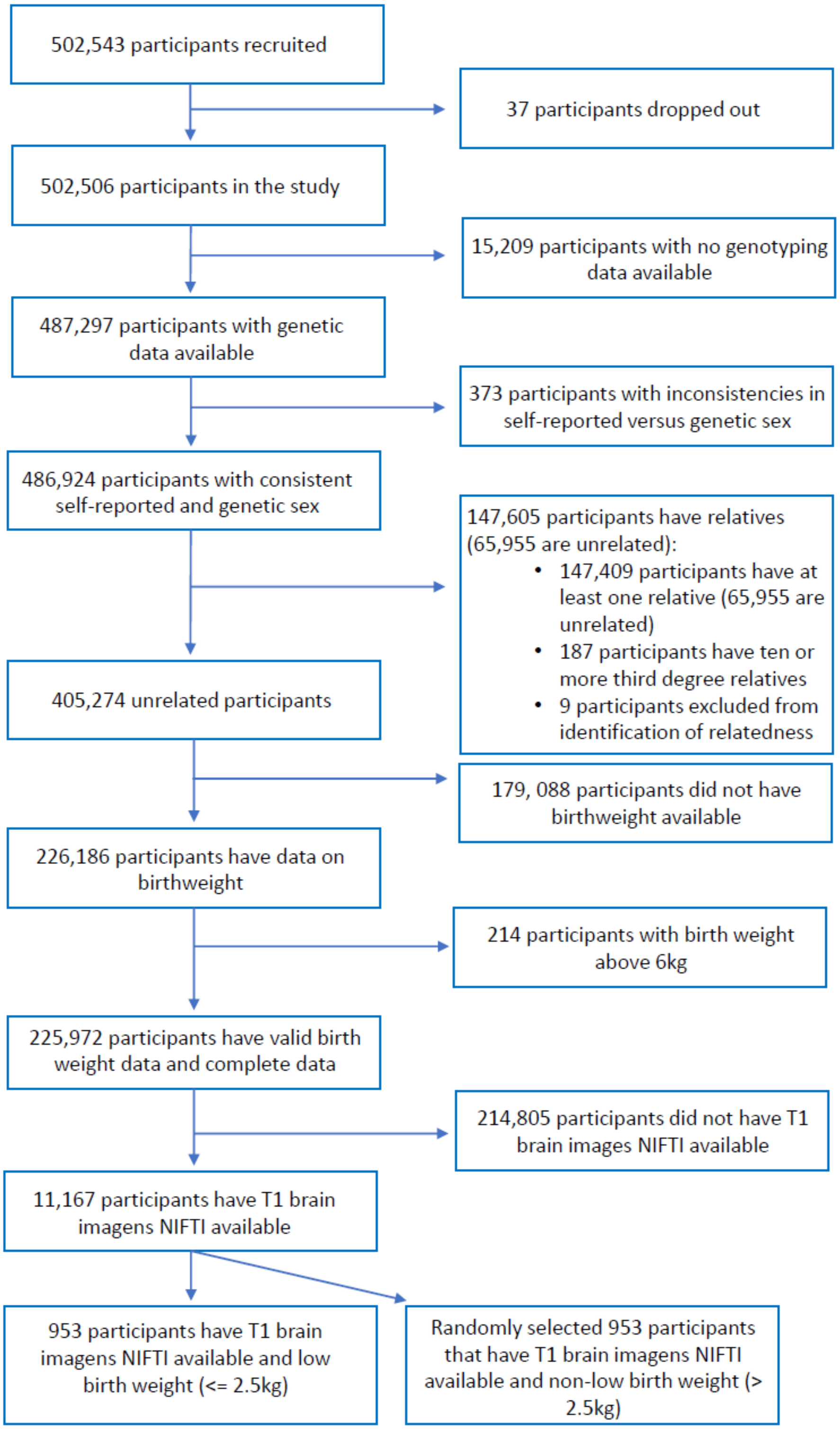

Supplementary Figure 1. UK Biobank Sample size block scheme for each inclusion and exclusion criteria applied. Block scheme depicting each step of inclusion and exclusion criteria applied to the original sample (N=502,543) until reaching final sample size for main hypothesis testing (N=225,972) and final sample size for parallel ICA analysis group of low birth weight (N=953) and non-low birth weight (N=953).

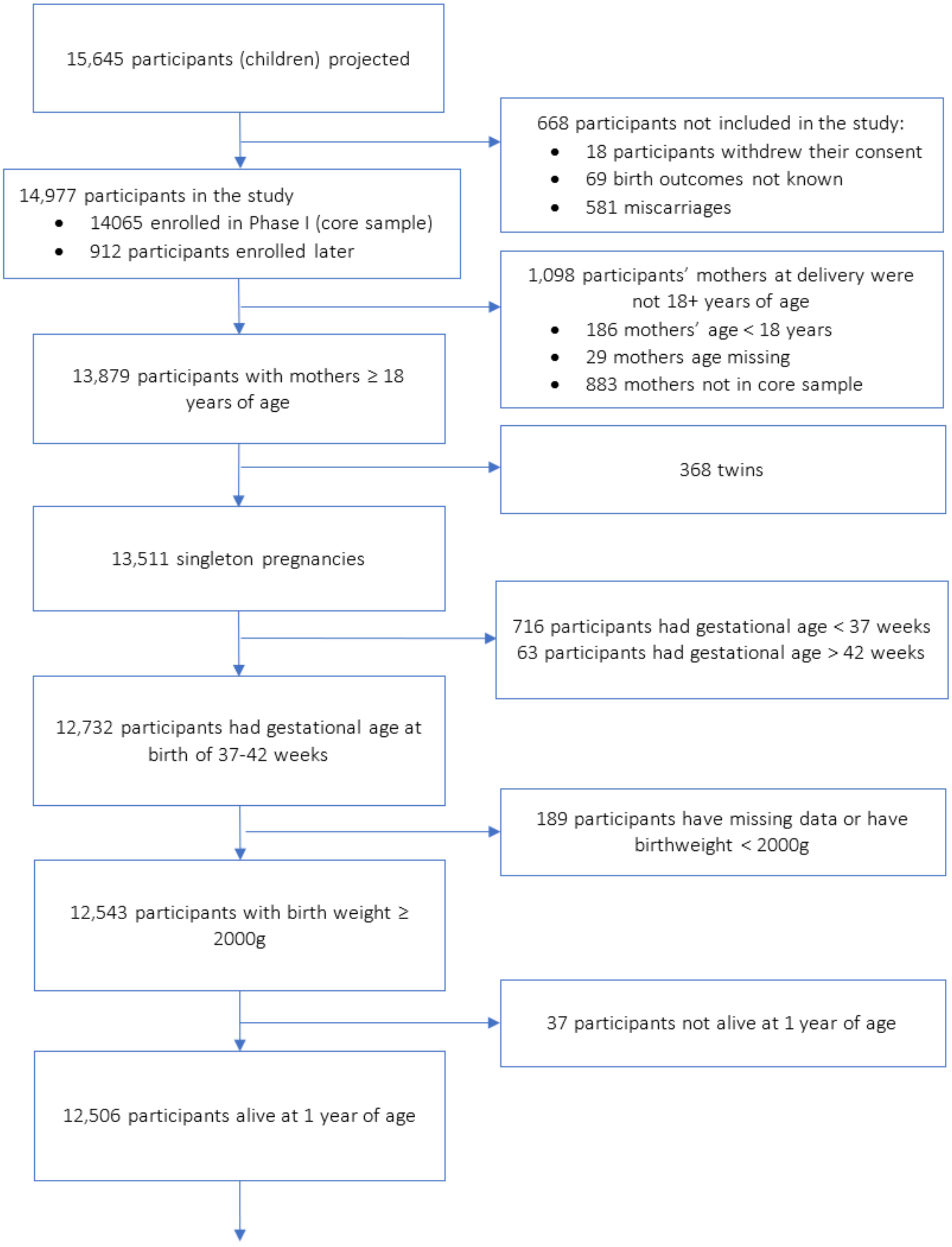
15,645 participants (children) projected
668 participants not included in the study:
18 participants withdrew their consent
69 birth outcomes not known
581 miscarriages
14,977 participants in the study
14065 enrolled in Phase I (core sample)
912 participants enrolled later
1,098 participants' mothers at delivery were not 18+ years of age
186 mothers' age < 18 years
29 mothers age missing
883 mothers not in core sample
13,879 participants with mothers ≥ 18 years of age
368 twins
13,511 singleton pregnancies
716 participants had gestational age < 37 weeks
63 participants had gestational age > 42 weeks
12,732 participants had gestational age at birth of 37-42 weeks
189 participants have missing data or have birthweight < 2000g
12,543 participants with birth weight ≥ 2000g
37 participants not alive at 1 year of age
12,506 participants alive at 1 year of age

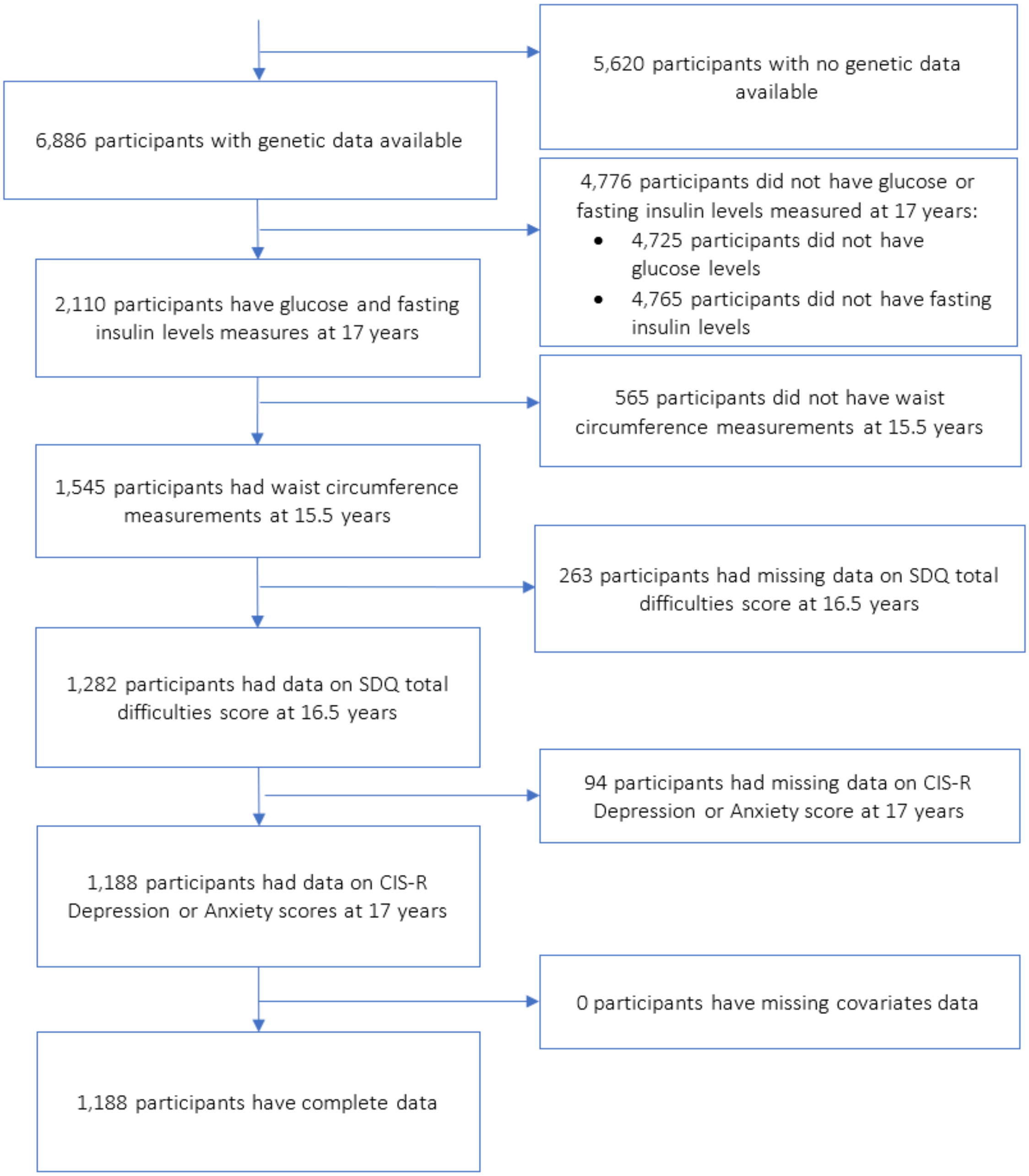

Supplementary Figure 2. ALSPAC Sample size block scheme for each inclusion and exclusion criteria applied Block scheme depicting each step of inclusion and exclusion criteria applied to the original sample (N=15,645) until reaching final sample size for main hypothesis testing (N=1,188).

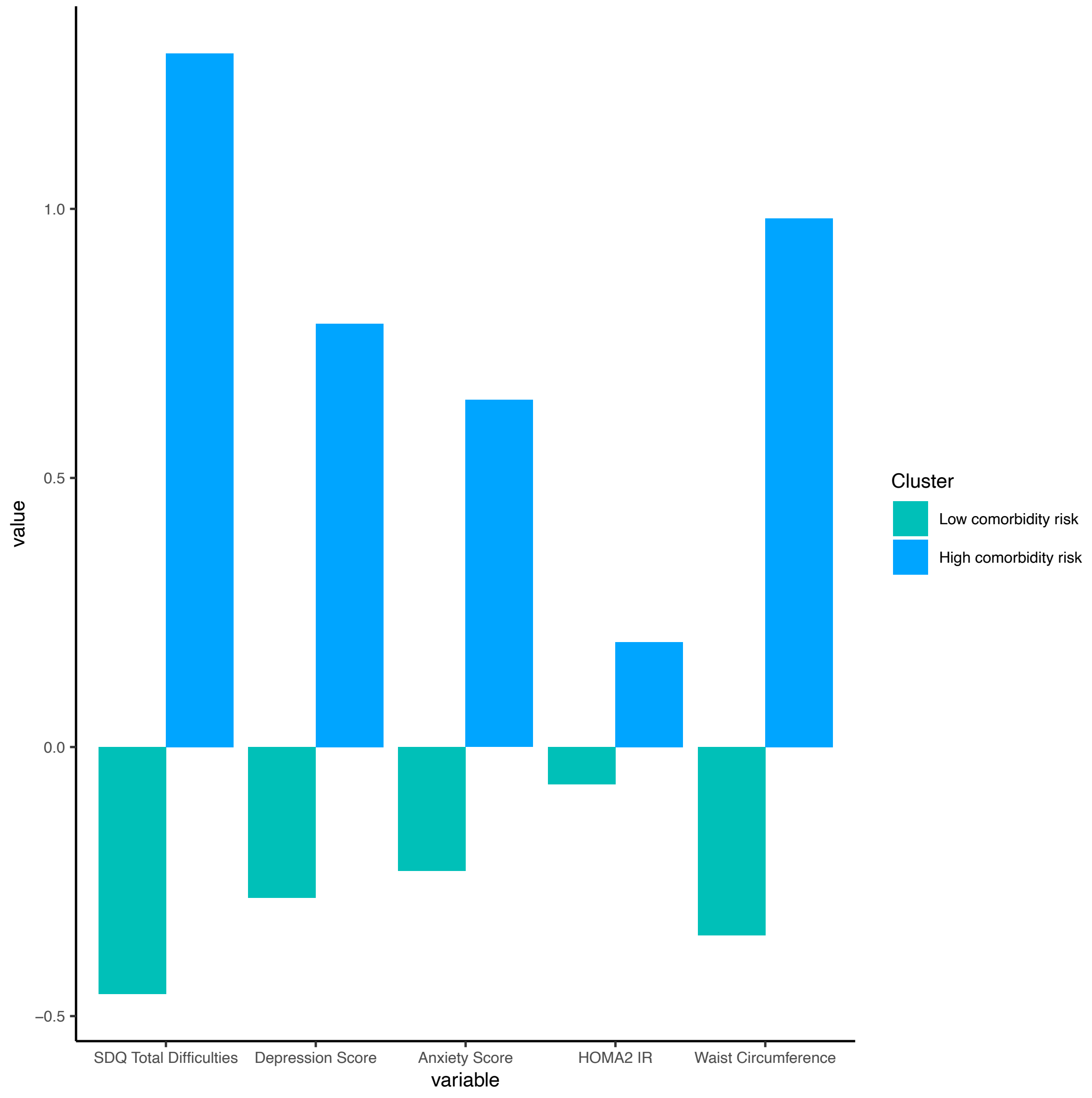

Supplementary Figure 3. Comorbidity risk clusters – ALSPAC. Cluster 1 – Low Comorbidity Risk (N=876) and Cluster 2 – High Comorbidity Risk (N=312) mean z score values for the variables considered in the cluster analysis. Cluster high comorbidity risk represents the psychiatric and metabolic comorbidity risk profile in adolescents. SDQ Total Difficulties is a score from the Strengths & Difficulties Questionnaire (SDQ). Depression and anxiety scores were computed from the Computerized Interview Schedule – Revised (CIS-R). Homeostatic Model Assessment of Insulin Resistance (HOMA-IR) was computed using the HOMA2 calculation tool. Waist circumference was measured in centimeters. Clustering of these variables was performed using mclust package in R.

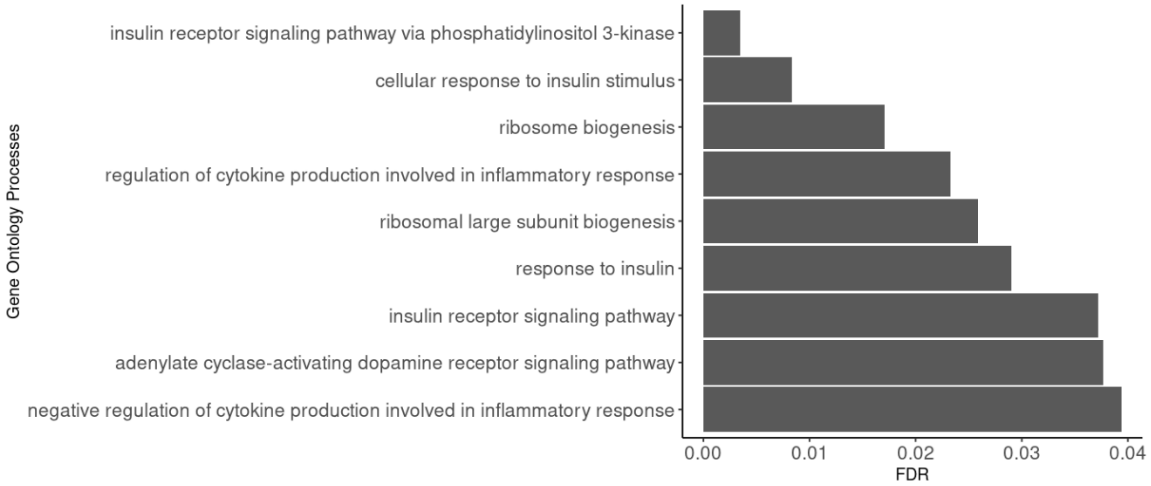

Supplementary Figure 4. Striatal SLC6A3 gene network enrichment analysis. Gene ontology processes related to genes included in striatal SLC6A3 gene network. Enrichment was performed using MetaCore®. The significance was considered for the false discovery rate (FDR) adjusted p-value <0.05.

Supplementary Table 1. Genes co-expressed with the SLC6A3 in mice striatum data, used to calculate striatum *SLC6A3* ePGS genetic score.

Mouse gene	Mouse Ensembl gene ID	Human gene	Human Ensembl gene ID	Description
Rnf165	ENSMUSG00000025427	RNF165	ENSG00000141622	ring finger protein 165 [Source:HGNC Symbol;Acc:HGNC:31696]
Rps21	ENSMUSG00000039001	RPS21	ENSG00000171858	ribosomal protein S21 [Source:HGNC Symbol;Acc:HGNC:10409]
Ipmk	ENSMUSG00000060733	IPMK	ENSG00000151151	inositol polyphosphate multikinase [Source:HGNC Symbol;Acc:HGNC:20739]
Mllt3	ENSMUSG00000028496	MLLT3	ENSG00000171843	MLLT3 super elongation complex subunit [Source:HGNC Symbol;Acc:HGNC:7136]
Hnrnpk	ENSMUSG00000021546	HNRNPK	ENSG00000165119	heterogeneous nuclear ribonucleoprotein K [Source:HGNC Symbol;Acc:HGNC:5044]
Rsl1d1	ENSMUSG00000005846	RSL1D1	ENSG00000171490	ribosomal L1 domain containing 1 [Source:HGNC Symbol;Acc:HGNC:24534]
Gfra2	ENSMUSG00000022103	GFRA2	ENSG00000168546	GDNF family receptor alpha 2 [Source:HGNC Symbol;Acc:HGNC:4244]
Nptxr	ENSMUSG00000022421	NPTXR	ENSG00000221890	neuronal pentraxin receptor [Source:HGNC Symbol;Acc:HGNC:7954]
Tgif2	ENSMUSG00000062175	TGIF2	ENSG00000118707	TGFB induced factor homeobox 2 [Source:HGNC Symbol;Acc:HGNC:15764]
Jarid2	ENSMUSG00000038518	JARID2	ENSG00000008083	jumonji and AT-rich interaction domain containing 2 [Source:HGNC Symbol;Acc:HGNC:6196]
Bmf	ENSMUSG00000040093	BMF	ENSG00000104081	Bcl2 modifying factor [Source:HGNC Symbol;Acc:HGNC:24132]
6820408 C15Rik	ENSMUSG00000032680	C20orf96	ENSG00000196476	chromosome 20 open reading frame 96 [Source:HGNC Symbol;Acc:HGNC:16227]
Rpl14	ENSMUSG00000025794	RPL14	ENSG00000188846	ribosomal protein L14 [Source:HGNC Symbol;Acc:HGNC:10305]
Stk32a	ENSMUSG00000039954	STK32A	ENSG00000169302	serine/threonine kinase 32A [Source:HGNC Symbol;Acc:HGNC:28317]
Cdh10	ENSMUSG00000022321	CDH10	ENSG00000040731	cadherin 10 [Source:HGNC Symbol;Acc:HGNC:1749]
Sdc3	ENSMUSG00000025743	SDC3	ENSG00000162512	syndecan 3 [Source:HGNC Symbol;Acc:HGNC:10660]
Mkrn1	ENSMUSG00000029922	MKRN1	ENSG00000133606	makorin ring finger protein 1 [Source:HGNC Symbol;Acc:HGNC:7112]
Bbc3	ENSMUSG00000002083	BBC3	ENSG00000105327	BCL2 binding component 3 [Source:HGNC Symbol;Acc:HGNC:17868]
Tubb2a	ENSMUSG00000058672	TUBB2A	ENSG00000137267	tubulin beta 2A class IIa [Source:HGNC Symbol;Acc:HGNC:12412]
Bcl11a	ENSMUSG00000000861	BCL11A	ENSG00000119866	BAF chromatin remodeling complex subunit BCL11A [Source:HGNC Symbol;Acc:HGNC:13221]
Rpl37a	ENSMUSG00000046330	RPL37A	ENSG00000197756	ribosomal protein L37a [Source:HGNC Symbol;Acc:HGNC:10348]
Cotl1	ENSMUSG00000031827	COTL1	ENSG00000103187	coactosin like F-actin binding protein 1 [Source:HGNC Symbol;Acc:HGNC:18304]
Arl4c	ENSMUSG00000049866	ARL4C	ENSG00000188042	ADP ribosylation factor like GTPase 4C [Source:HGNC Symbol;Acc:HGNC:698]
Rpl27	ENSMUSG00000063316	RPL27	ENSG00000131469	ribosomal protein L27 [Source:HGNC Symbol;Acc:HGNC:10328]
Rrm1	ENSMUSG00000030978	RRM1	ENSG00000167325	ribonucleotide reductase catalytic subunit M1 [Source:HGNC Symbol;Acc:HGNC:10451]
Rpl30	ENSMUSG00000058600	RPL30	ENSG00000156482	ribosomal protein L30 [Source:HGNC Symbol;Acc:HGNC:10333]
Gbx2	ENSMUSG00000034486	GBX2	ENSG00000168505	gastrulation brain homeobox 2 [Source:HGNC Symbol;Acc:HGNC:4186]
Cux1	ENSMUSG00000029705	CUX1	ENSG00000257923	cut like homeobox 1 [Source:HGNC Symbol;Acc:HGNC:2557]
Dcaf12	ENSMUSG00000028436	DCAF12	ENSG00000198876	DDB1 and CUL4 associated factor 12 [Source:HGNC Symbol;Acc:HGNC:19911]
Klhl8	ENSMUSG00000029312	KLHL8	ENSG00000145332	kelch like family member 8 [Source:HGNC Symbol;Acc:HGNC:18644]
Zfy1	ENSMUSG00000053211	ZNF586	ENSG00000083828	zinc finger protein 586 [Source:HGNC Symbol;Acc:HGNC:25949]

Zfy1	ENSMUSG00000053211	ZNF480	ENSG00000198464	zinc finger protein 480 [Source:HGNC Symbol;Acc:HGNC:23305]
Zfy1	ENSMUSG00000053211	ZNF548	ENSG00000188785	zinc finger protein 548 [Source:HGNC Symbol;Acc:HGNC:26561]
Stag1	ENSMUSG00000037286	STAG1	ENSG00000118007	stromal antigen 1 [Source:HGNC Symbol;Acc:HGNC:11354]
Apc	ENSMUSG00000005871	APC	ENSG00000134982	APC regulator of WNT signaling pathway [Source:HGNC Symbol;Acc:HGNC:583]
Zfy1	ENSMUSG00000053211	ZNF549	ENSG00000121406	zinc finger protein 549 [Source:HGNC Symbol;Acc:HGNC:26632]
Zfy1	ENSMUSG00000053211	ZNF134	ENSG00000213762	zinc finger protein 134 [Source:HGNC Symbol;Acc:HGNC:12918]
Zfy1	ENSMUSG00000053211	ZNF304	ENSG00000131845	zinc finger protein 304 [Source:HGNC Symbol;Acc:HGNC:13505]
Zfy1	ENSMUSG00000053211	ZNF154	ENSG00000179909	zinc finger protein 154 [Source:HGNC Symbol;Acc:HGNC:12939]
Zfy1	ENSMUSG00000053211	ZNF793	ENSG00000188227	zinc finger protein 793 [Source:HGNC Symbol;Acc:HGNC:33115]
Zfy1	ENSMUSG00000053211	ZNF772	ENSG00000197128	zinc finger protein 772 [Source:HGNC Symbol;Acc:HGNC:33106]
Ptpn9	ENSMUSG00000032290	PTPN9	ENSG00000169410	protein tyrosine phosphatase non-receptor type 9 [Source:HGNC Symbol;Acc:HGNC:9661]
Hnrnpa1	ENSMUSG00000046434	HNRNPA1	ENSG00000135486	heterogeneous nuclear ribonucleoprotein A1 [Source:HGNC Symbol;Acc:HGNC:5031]
Myd88	ENSMUSG00000032508	MYD88	ENSG00000172936	MYD88 innate immune signal transduction adaptor [Source:HGNC Symbol;Acc:HGNC:7562]
Brd4	ENSMUSG00000024002	BRD4	ENSG00000141867	bromodomain containing 4 [Source:HGNC Symbol;Acc:HGNC:13575]
Ube2i	ENSMUSG00000015120	UBE2I	ENSG00000103275	ubiquitin conjugating enzyme E2 I [Source:HGNC Symbol;Acc:HGNC:12485]
Apc2	ENSMUSG00000020135	APC2	ENSG00000115266	APC regulator of WNT signaling pathway 2 [Source:HGNC Symbol;Acc:HGNC:24036]
Trh	ENSMUSG00000005892	TRH	ENSG00000170893	thyrotropin releasing hormone [Source:HGNC Symbol;Acc:HGNC:12298]
Pklr	ENSMUSG00000041237	PKLR	ENSG00000143627	pyruvate kinase L/R [Source:HGNC Symbol;Acc:HGNC:9020]
Krt18	ENSMUSG00000023043	KRT18	ENSG00000111057	keratin 18 [Source:HGNC Symbol;Acc:HGNC:6430]
Hnrnpr	ENSMUSG00000066037	HNRNPR	ENSG00000125944	heterogeneous nuclear ribonucleoprotein R [Source:HGNC Symbol;Acc:HGNC:5047]
Agrn	ENSMUSG00000041936	AGRN	ENSG00000188157	agrin [Source:HGNC Symbol;Acc:HGNC:329]
Cct3	ENSMUSG00000001416	CCT3	ENSG00000163468	chaperonin containing TCP1 subunit 3 [Source:HGNC Symbol;Acc:HGNC:1616]
Rpl26	ENSMUSG00000060938	RPL26	ENSG00000161970	ribosomal protein L26 [Source:HGNC Symbol;Acc:HGNC:10327]
Eef1b2	ENSMUSG00000025967	EEF1B2	ENSG00000114942	eukaryotic translation elongation factor 1 beta 2 [Source:HGNC Symbol;Acc:HGNC:3208]
Gfer	ENSMUSG00000040888	GFER	ENSG00000127554	growth factor, augmenter of liver regeneration [Source:HGNC Symbol;Acc:HGNC:4236]
Ephb2	ENSMUSG00000028664	EPHB2	ENSG00000133216	EPH receptor B2 [Source:HGNC Symbol;Acc:HGNC:3393]
Zfp454	ENSMUSG00000048728	ZNF454	ENSG00000178187	zinc finger protein 454 [Source:HGNC Symbol;Acc:HGNC:21200]
Rps20	ENSMUSG00000028234	RPS20	ENSG00000008988	ribosomal protein S20 [Source:HGNC Symbol;Acc:HGNC:10405]
Mllt11	ENSMUSG00000053192	MLLT11	ENSG00000213190	MLLT11 transcription factor 7 cofactor [Source:HGNC Symbol;Acc:HGNC:16997]
Pias4	ENSMUSG00000004934	PIAS4	ENSG00000105229	protein inhibitor of activated STAT 4 [Source:HGNC Symbol;Acc:HGNC:17002]
Rps13	ENSMUSG00000090862	RPS13	ENSG00000110700	ribosomal protein S13 [Source:HGNC Symbol;Acc:HGNC:10386]
Grwd1	ENSMUSG00000053801	GRWD1	ENSG00000105447	glutamate rich WD repeat containing 1 [Source:HGNC Symbol;Acc:HGNC:21270]
Ambra1	ENSMUSG00000040506	AMBRA1	ENSG00000110497	autophagy and beclin 1 regulator 1 [Source:HGNC Symbol;Acc:HGNC:25990]

Pik3ca	ENSMUSG00000027665	PIK3CA	ENSG00000121879	phosphatidylinositol-4,5-bisphosphate 3-kinase catalytic subunit alpha [Source:HGNC Symbol;Acc:HGNC:8975]
Rpl13	ENSMUSG00000000740	RPL13	ENSG00000167526	ribosomal protein L13 [Source:HGNC Symbol;Acc:HGNC:10303]
Gnb1	ENSMUSG00000029064	GNB1	ENSG00000078369	G protein subunit beta 1 [Source:HGNC Symbol;Acc:HGNC:4396]

Supplementary Table 1. 558 unique mouse genes with absolute correlation index >= 0.5 were retained from GeneNetwork. These genes were converted to 381 unique human genes. The 381 unique human genes were filtered using BrainSpan data (overly expressed striatum genes in all prenatal vs adult samples) resulting in 72 genes. Of these 72 genes, 67 were found in GTEx and our study sample and were used to calculate striatum *SLC6A3 ePGS.*

Supplementary Table 2. Means of variables used in the cluster analysis for the ALSPAC cohort.

	Cluster - Low comorbidity risk		Cluster – High comorbidity risk		P-value
	Mean / %	SE / N	Mean / %	SE / N	
SDQ Total Difficulties	5.02	0.14	6.77	0.26	<0.001
Depression Score	0	0	1.08	0.06	<0.001
Anxiety Score	0	0	0.88	0.06	<0.001
HOMA2-IR	0.81	0.01	1.08	0.05	<0.001
Waist Circumference	75.9	0.25	77.17	0.57	0.04
Sex -Male	49.5%	434	42%	131	0.03
zBMI	.26	0.03	0.31	0.06	0.45
Birth weight (kg)	3.515	0.015	3.49	0.025	0.44
	N=876		N=312		

Supplementary Table 3. Main effect of PRS on psychiatric and cardio-metabolic comorbidity.

		Main effect		
**UK Biobank**	**PRS scores**	***p***	**β**	**OR**
	Type 2 diabetes PRS_EPIC	< 0.001	0.073	1.076
	Major depression disorders PRS	< 0.001	0.065	1.068
**ALSPAC**	**PRS scores**	***p***	**β**	**OR**
	Type 2 diabetes PRS_EPIC	0.253	-0.076	0.927
	Major depression disorders PRS	0.882	0.020	1.020

Supplementary Table 4. Interaction effect between PRS and birth weight on psychiatric and cardio-metabolic comorbidity.

		Interaction effect		
**UK Biobank**	**PRS scores**	***p***	**β**	**OR**
	Type 2 diabetes PRS_EPIC	0.490	0.014	1.014
	Major depression disorders PRS	0.608	-0.011	0.989
**ALSPAC**	**PRS scores**	***p***	**β**	**OR**
	Type 2 diabetes PRS_EPIC	0.461	0.112	1.119
	Major depression disorders PRS	0.798	-0.078	0.925

Supplementary Table 5. Characteristics comparison between low vs randomly-selected non-low birth weight groups used in the parallel ICA analysis

UK Biobank – Low vs randomly-selected Non-low Birth Weight group (n=1906)					
Characteristics	Low Birth Weight (n =953)		Non-low Birth Weight (n = 953)		P-value
	Mean or percentage	SD or N	Mean or percentage	SD or N	
Sex - Male	34.7%	331	44.7%	426	P<0.001
Birth weight (grams)	2129	376	3464	493	P<0.001
Completed full-time education at 14-years of age or younger	0.7%	4	0.9%	5	0.97
Age at recruitment (years)	54.95	7.37	53.8	7.45	P<0.001
Townsend deprivation index at recruitment	-1.79	2.75	-2.02	2.69	0.06
BMI at recruitment	26.83	4.45	26.52	4.24	0.11
DAT1 STR ePGS	0.02	0.95	0.07	0.96	0.29

Supplementary Table 6. Characteristics comparison between randomly-selected non-low birth weight group used in the parallel ICA analysis vs full sample of non-low birth weight individuals with MRI data available in the UK Biobank

UK Biobank – randomly-selected non-low birth weight group vs rest of non-low birth weight group (n=10214)					
Characteristics	Randomly-selected Non-low Birth Weight (n =953)		Full sample of Non-low Birth Weight (n = 9261)		P-value
	Mean or percentage	SD or N	Mean or percentage	SD or N	
Sex - Male	44.7%	426	42.3%	3920	0.17
Birth weight (grams)	3464	493	3460	495	0.79
Completed full-time education at 14-years of age or younger	0.9%	5	0.5%	27	0.4
Age at recruitment (years)	53.8	7.45	53.75	7.38	0.85
Townsend deprivation index at recruitment	-2.02	2.69	-2.07	2.59	0.617
BMI at recruitment	26.52	4.24	26.51	4.34	0.956
DAT1 STR ePGS	0.07	0.96	0.05	0.96	0.607

Supplementary Table 7. Subset of significant SNPs related to gray matter density variations according to pICA analysis.

Significant SNPs	
SNP	Z Score
rs34605159	12.99878
rs7259029	-11.3745
rs139012629	11.11315
rs115385848	-9.25124
rs116962884	8.970151
rs11704200	8.799921
rs184236146	-7.28849
rs6737722	7.180836
rs112664980	5.614825
rs56988084	5.071326
rs2556378	-5.00336
rs76380377	-4.92952
rs67621412	4.895654
rs72795692	4.635065
rs6947066	-4.32333
rs1169557	4.131825
rs3762272	-3.79013
rs372339543	-3.7461
rs58399787	-3.63446
rs2966318	-3.50433
rs2967848	3.478973
rs140310740	3.390835
rs70953651	3.35623
rs149133487	-3.20932
rs71526493	-3.17741
rs3128100	3.123754
rs2967871	-3.06511
rs117055030	2.941269
rs1536096	2.915037
rs11689362	2.813534
rs3121575	-2.7558
rs57843539	-2.61199
rs11707190	2.509964
rs2048008	2.500896

Supplementary References

1 Bycroft, C. *et al.* The UK Biobank resource with deep phenotyping and genomic data. *Nature* **562**, 203 (2018).

2 Galinsky, K. J. *et al.* Fast Principal-Component Analysis Reveals Convergent Evolution of ADH1B in Europe and East Asia. *Am J Hum Genet* **98**, 456-472 (2016). https://doi.org/10.1016/j.ajhg.2015.12.022

3 Richmond, R. C. *et al.* Using genetic variation to explore the causal effect of maternal pregnancy adiposity on future offspring adiposity: a Mendelian randomisation study. *PLoS medicine* **14** (2017).

4 Patterson, N., Price, A. L. & Reich, D. Population structure and eigenanalysis. *PLoS genetics* **2** (2006).

5 Price, A. L. *et al.* Principal components analysis corrects for stratification in genome-wide association studies. *Nature genetics* **38**, 904-909 (2006).

6 Chang, C. C. *et al.* Second-generation PLINK: rising to the challenge of larger and richer datasets. *Gigascience* **4**, s13742-13015-10047-13748 (2015).

7 Miller, J. A. *et al.* Transcriptional landscape of the prenatal human brain. *Nature* **508**, 199 (2014).

8 R, C. T. *R: A language and environment for statistical computing.*, (R Foundation for Statistical Computing, 2014).

9 Galili, T., O'Callaghan, A., Sidi, J. & Sievert, C. heatmaply: an R package for creating interactive cluster heatmaps for online publishing. *Bioinformatics* **34**, 1600-1602 (2018). https://doi.org/10.1093/bioinformatics/btx657

10 Organization, W. H. (2004).

11 Goodman, R. The Strengths and Difficulties Questionnaire: a research note. *Journal of child psychology and psychiatry* **38**, 581-586 (1997).

12 Lewis, G. Assessing psychiatric disorder with a human interviewer or a computer. *Journal of Epidemiology & Community Health* **48**, 207-210 (1994).

13 Wallace, T. M., Levy, J. C. & Matthews, D. R. Use and abuse of HOMA modeling. *Diabetes care* **27**, 1487-1495 (2004).

14 Alfaro-Almagro, F. *et al.* Image processing and Quality Control for the first 10,000 brain imaging datasets from UK Biobank. *Neuroimage* **166**, 400-424 (2018).

15 Kang, J. *et al.* Ribosomal proteins and human diseases: molecular mechanisms and targeted therapy. *Signal Transduct Target Ther* **6**, 323 (2021). https://doi.org/10.1038/s41392-021-00728-8

16 Smagin, D. A. *et al.* Dysfunction in Ribosomal Gene Expression in the Hypothalamus and Hippocampus following Chronic Social Defeat Stress in Male Mice as Revealed by RNA-Seq. *Neural Plast* **2016**, 3289187 (2016). https://doi.org/10.1155/2016/3289187

17 Katzav, A. *et al.* Induction of autoimmune depression in mice by anti-ribosomal P antibodies via the limbic system. *Arthritis Rheum* **56**, 938-948 (2007). https://doi.org/10.1002/art.22419

18 Murakami, T. *et al.* Cutting Edge: G Protein Subunit β 1 Negatively Regulates NLRP3 Inflammasome Activation. *J Immunol* **202**, 1942-1947 (2019). https://doi.org/10.4049/jimmunol.1801388

19 Roohi, E., Jaafari, N. & Hashemian, F. On inflammatory hypothesis of depression: what is the role of IL-6 in the middle of the chaos? *Journal of Neuroinflammation* **18**, 45 (2021). https://doi.org/10.1186/s12974-021-02100-7
20 Karhunen, V. *et al.* The interplay between inflammatory cytokines and cardiometabolic disease: bi-directional mendelian randomisation study. *BMJ Medicine* **2**, e000157 (2023). https://doi.org/10.1136/bmjmed-2022-000157
21 Dreyfuss, G., Kim, V. N. & Kataoka, N. Messenger-RNA-binding proteins and the messages they carry. *Nature reviews Molecular cell biology* **3**, 195-205 (2002).
22 Dalla Costa, I. *et al.* The functional organization of axonal mRNA transport and translation. *Nature Reviews Neuroscience*, 1-15 (2020).
23 Choi, C. S. *et al.* Effects of atomoxetine on hyper-locomotive activity of the prenatally valproate-exposed rat offspring. *Biomolecules & therapeutics* **22**, 406 (2014).
24 Jijun, L. *et al.* Abnormal expression of dopamine and serotonin transporters associated with the pathophysiologic mechanism of Tourette syndrome. *Neurology India* **58**, 523 (2010).
25 Sun, Z. *et al.* Behavioral changes and neuronal damage in rhesus monkeys after 10 weeks of ketamine administration involve prefrontal cortex dopamine D2 receptor and dopamine transporter. *Neuroscience* **415**, 97-106 (2019).
26 Ciliax, B. J. *et al.* Immunocytochemical localization of the dopamine transporter in human brain. *Journal of Comparative Neurology* **409**, 38-56 (1999).
27 Bagalkot, T. R. *et al.* Dopamine transporter localization in medial forebrain bundle axons indicates its long-range transport primarily by membrane diffusion with a limited contribution of vesicular traffic on retromer-positive compartments. *Journal of Neuroscience* **41**, 234-250 (2021).
28 Schüring, A. N. *et al.* Role of syndecan-3 polymorphisms in obesity and female hyperandrogenism. *J Mol Med (Berl)* **87**, 1241-1250 (2009). https://doi.org/10.1007/s00109-009-0529-1
29 Mathieu, P., Lemieux, I. & Després, J. P. Obesity, inflammation, and cardiovascular risk. *Clinical pharmacology & therapeutics* **87**, 407-416 (2010).
30 Shoelson, S. E., Herrero, L. & Naaz, A. Obesity, inflammation, and insulin resistance. *Gastroenterology* **132**, 2169-2180 (2007).
31 Vosberg, D. E., Leyton, M. & Flores, C. The Netrin-1/DCC guidance system: dopamine pathway maturation and psychiatric disorders emerging in adolescence. *Mol Psychiatry* **25**, 297-307 (2020). https://doi.org/10.1038/s41380-019-0561-7
32 Hoops, D. & Flores, C. Making dopamine connections in adolescence. *Trends in neurosciences* **40**, 709-719 (2017).
33 Batra, A. *et al.* Relationship between insulin and Netrin-1/DCC guidance cue pathway regulation in the prefrontal cortex of rodents exposed to prenatal dietary restriction. *Journal of Developmental Origins of Health and Disease* **14**, 501-507 (2023). https://doi.org/10.1017/S204017442300017X
34 Wang, C., Geng, B., Cui, Q., Guan, Y. & Yang, J. Intracellular and extracellular adenosine triphosphate in regulation of insulin secretion from pancreatic β cells (β). *J Diabetes* **6**, 113-119 (2014). https://doi.org/10.1111/1753-0407.12098

35 Patterson, J. N. *et al.* Mitochondrial metabolism of pyruvate is essential for regulating glucose-stimulated insulin secretion. *Journal of Biological Chemistry* **289**, 13335-13346 (2014).
